# New Insight and Confrontation of the Internal Structure and Sensilla of the Mouthparts of Cicadomorpha (Insecta: Hemiptera)

**DOI:** 10.3390/insects16101026

**Published:** 2025-10-04

**Authors:** Jolanta Brożek, Piotr Wegierek, Mick Webb, Adam Stroiński

**Affiliations:** 1 Faculty of Natural Sciences, Institute Biology, Biotechnology and Environmental Protection, University of Silesia in Katowice, 40-007 Katowice, Poland; jolanta.brozek@us.edu.pl (J.B.); piotr.wegierek@us.edu.pl (P.W.); 2 Department of Life Sciences, Natural History Museum, Cromwell Road, London SW7 5BD, UK; m.webb@nhm.ac.uk; 3 Museum and Institute of Zoology, Polish Academy of Sciences, 00-818 Warsaw, Poland

**Keywords:** morphology, sensilla types and function, internal of structure mouthparts, SEM techniques

## Abstract

**Simple Summary:**

The microstructural examination of Cicadomorpha mouthparts reveals a similar morphological framework in the studied species, essential for sap feeding from floem, xylem, or epidermis cells. The consistent presence of the dual interlocking maxillary stylet system and overall mouthpart architecture underscores evolutionary stability within the group. In contrast, variations in mandibular barbs, salivary canal closure types, and the diversity of sensilla on the labial tip (contact chemoreceptor, gustatory, olfactory, and thermo-higroreceptors) suggest functional adaptations to different host plant interactions. These findings contribute to a deeper understanding of the morphology and sensory differences in Cicadomorpha.

**Abstract:**

This study presents detailed microstructural observations of the mouthparts and sensory organs of adult cicadomorphan species, obtained using scanning electron microscopy (SEM). Despite microstructural variation, the overall morphology of the mouthparts, comprising a three-segmented labium and a bundle of interlocking stylets (maxillae and mandibles), is highly conserved across species, supporting its evolutionary significance in sap feeding from floem, xylem, or epidermis cells. Variations in the number and shape of mandibular stylet barbs likely reflect adaptations to different host plant tissues. The presence of an identical dual interlocking system between the maxillary stylets, which is found consistently across taxa, enhances functional stability during feeding and indicates a conserved mechanism among cicadomorphans. The species studied exhibit two distinct types of salivary canal closure: hooked and T-shaped. The latter potentially represents a state linked to specialised feeding strategies, such as sap xylem feeding. On the labial tip, there are different shapes of the anterior sensory fields. This area hosts a complex array of sensilla of different numbers, including gustatory (sensilla peg, PS1 and PS2, basiconica, BS3, double basiconica, DB), olfactory (finger–like, FLS) and thermo-hygroreceptive (sensillum dome-shaped, DS, and coeloconicum, CS) types, which facilitate host detection and feeding site selection. In the posterior sensory field, sensilla contact-chemosensory (sensilla basiconica, BS1 and BS2, and sensillum trichoideum, TS) are present. Mechanosensilla chaetica (CH1–CH3) are widely distributed on the last labial segment and may contribute to labium positioning. These findings emphasise the presence of both conserved and specialised morphological traits reflecting evolutionary and ecological diversification within Cicadomorpha.

## 1. Introduction

The order Hemiptera comprises five extant suborders: Sternorrhyncha, Fulgoromorpha, Cicadomorpha, Coleorrhyncha, and Heteroptera [1,2]. Hemiptera is widely recognised as a monophyletic group, primarily due to the unique morphology of their mouthparts. The mandibles and maxillary laciniae are modified into concentric stylets: the mandibular stylets envelop the maxillary stylets, which internally form the food and salivary canals. These structures are enclosed within a multi-segmented labium, notably lacking maxillary and labial palpi [3,4,5,6]. The suborder Cicadomorpha includes the superfamilies Cicadoidea (cicadas), Membracoidea (leafhoppers and treehoppers), and Cercopoidea (spittlebugs) [7,8,9,10].

The evolutionary diversification of insect mouthparts has been largely influenced by the availability and variety of food sources [11]. Among hemipterans, plant sap serves as the primary nutrient source in Sternorrhyncha, Fulgoromorpha, and Cicadomorpha, where sap feeding is obligate. In these groups—and in most Heteroptera—plant sap is extracted using highly specialised piercing–sucking mouthparts that facilitate either active or passive ingestion [4,12].

The mouthpart forms a rostrum, a tubular structure derived mainly from modifications of the labium (lower lip) and, to a lesser extent, the labrum (upper lip). The conical labrum is typically suspended from the anteclypeus and covers the base of the labium, which houses the maxillary and mandibular stylets. Regarding labrum morphology in cicadomorphans, no significant differences in shape or in the presence of non-sensory protuberances—such as acanthae and scale-like microtrichia—have been observed between *Homalodisca coagulata* [13] and *Nacolus tuberculatus* [14]. Microtrichia are present in ten genera of Membracidae, while acanthae are observed in only two. In most species, the labrum is rather covered randomly by hair-like mechanosensilla [15].

In leafhoppers (Cicadellidae), the labium is a long, cylindrical structure, typically divided into four segments. The postlabium consists of a membranous basal segment with two lateral sclerites, while the remaining three longitudinal segments form the prelabium [16]. However, many authors consider the labium as three-segmented [13,14,15,17,18]. The anterodorsal surface of the labium is grooved and houses the stylet bundle. The mandibular stylets closely surround the maxillary stylets along most of their length, except proximally [19,20].

Morphometric analyses show that in leafhoppers, the mandibles are approximately 74–79% the length of the maxillae [16,21,22,23], while in planthoppers, they reach 93–99% [20]. The mandibular stylets usually terminate in serrated, pointed tips. For instance, *Zyginidia pullula* (Boheman, 1845) has 9–10 external dentations; *Hebata vitis* (Göthe, 1875), 10–11; and *Graphocephala fennahi* Young, 1977, 3–4 prominent dentations followed by ~20 smaller projections [16]. In *Macrosteles fascifrons* (Stål, 1858) and *Eupteryx melisae* Curtis, 1837, the distal tips bear barb-like ridges on the outer convex surface [19], a trait also observed in *H. coagulata* [13]. In *Psammotettix alienus* (Dahlbom, 1850), additional teeth-like projections are located along the inner edges of the mandibular stylets [24]. According to Carlson et al. [15], mandibular stylet morphology remains consistent across six Membracidae genera, with all exhibiting serrated mandibles and a row of recurved barbs along the inner margin. Mandibular stylets possess a thin, inconspicuous apodeme-like process arising from the inner basal angle, connected to the posterior end of the lora by the mandibular lever, which inserts at their basal outer edge [16]. The mandibular stylets alternately penetrate shallowly, whereas the maxillary stylets protract deeper into plant tissue [20]. The latter play a primary role in deep tissue penetration due to their smooth, curved structure, acute apices, and capacity for deflection during insertion [21,25]. In six Membracidae genera, Carlson et al. [15] reported uniform morphology of the maxillary stylets, which showed consistent curvature and a sharp apical structure.

Both mandibular and maxillary stylets contain dendritic canals, with few dendrites, which distally bifurcate mainly into two branches. Such sensory receptors within the main stylet canals have been demonstrated in several species, indicating proprioception innervation [23,26,27,28].

In cross-section, the stylet bundle comprises outer mandibular and inner maxillary stylets. The maxillary stylets interlock via a system of ridges and grooves, a configuration commonly reported across Hemiptera [4,20,23,29,30,31,32,33]. Emeljanov [34] noted that the maxillary stylets are twisted ~90° to a horizontal orientation in Cercopidae and Cicadidae and ~45° to a diagonal orientation in Cicadellidae.

The food canal opens anteriorly just before the stylet tips and posteriorly connects to a wide, cup-like cibarial (sucking) pump, which is linked to a muscle bundle. The salivary canal connects posteriorly to a hollow, cup-shaped salivary pump, which communicates ventrally with the salivary glands via a duct [16]. In Cicadellidae, the food canal is significantly larger than the salivary canal [29]. The elongate stylets permit dual canal function—upward sap flow and downward saliva flow. The feeding strategy, involving equal insertion of both mandibular and maxillary stylets into plant tissue, is typical of Aphididae [25]. In contrast, in Cicadomorpha and Fulgoromorpha, only initial shallow insertion involves mandibular stylets; subsequent deeper penetration is performed by maxillary stylets [20,21,35]. Other hemipteran groups exhibit variable stylet insertion behaviours [4,36].

The structural and functional aspects of the rostrum in Cicadomorpha and Fulgoromorpha have been well documented [20,21,22,23,25,37,38,39,40]. Within Cicadomorpha, the superfamilies Cicadoidea, Cercopoidea, and Membracoidea exhibit dietary diversity: cicadas and spittlebugs feed primarily on xylem, whereas most Membracoidea consume phloem or parenchyma, though some leafhoppers also utilise xylem [41]. The feeding behaviour of the enigmatic family Myerslopidae remains unclear. These ground-dwelling insects, often found in mossy habitats, are hypothesised to be bryophagous [42] or fungivorous [43], opposing earlier claims of predation [44]. *Mapuchea chilensis* (Nielson, 1996) appears to feed on roots and creeping stems of angiosperms. Salivary sheath traces observed in captivity terminate in vascular bundles, suggesting phloem sap feeding [45].

The labium in hemipteran and other insects is equipped with various types of sensilla, serving mechanoreceptive and chemoreceptive functions. A basal unit is a sensillum, which includes the cuticular structure, neuron(s), sheath cells, enclosed cavities, and associated structures. Mechanosensilla typically appear as hair-like projections or dome-shaped campaniform sensilla, both lacking pores and innervated by a single bipolar neuron terminating in a tubular body and are attached to the labium via a flexible socket [28,46,47]. Chemosensilla are categorised as olfactory (multiporous) or gustatory (uniporous) embedded in an inflexible socket [47]. While olfactory and gustatory sensilla are generally restricted to the palps in most insects [48], in Hemiptera, these sensilla are documented on the labial apex in many species of Fulgoromorpha and Heteroptera [20,49,50]. Some labial sensilla exhibit bimodal functions, combining chemo- and mechanosensory capabilities [20,51]. The contact chemoreceptive sensilla and gustatory sensilla in hemipterans are used for first probing the sap. Only the cibarial sensilla directly contact ingested food and recognise the proper taste of sap. The labium tip sensilla do not enter host tissue but monitor the external surface. Differences in the type of sensilla and distribution among 16 planthopper families have been documented [50], though such variation has not been observed in detail so far in a few species of Cicadomorpha that have been studied.

Morphological research into the mouthparts and fine structures has focused on only a few subfamilies of Cicadellidae (Cicadellinae, Deltocephalinae, and Typhlocybinae), a number of genera of Membracidae, and a single species of Cicadidae. Studies have revealed that the mouthparts exhibit structural similarities, with variations primarily observed in proportions and fine structural features, including the surface sculpturing and length of the stylets [13,15,16,52]. The presence and arrangement of various sensilla types and subtypes (chaetica, trichoid, basiconica, peg, digitoformia, and coeloconica) on the labial surfaces have also been documented in some cicadomorphan species [13,15,17,20,52,53,54]. Nevertheless, there is very little data on the detailed area of the sensory field, types, and the prefunction of the sensilla on the labial apex.

Further comparative studies of the mouthparts of a greater number of leafhopper species are required to reveal the extent to which the observed structures vary among the major lineages and to determine their significance in systematic and evolutionary studies, as well as in relation to feeding types.

The present study aims to describe the mouthpart morphology of selected representatives of Cicadomorpha and to provide comparative data analysis among these species within superfamilies. Through fine structural analysis, we will examine the components involved in plant penetration and feeding, focusing inter alia on sensilla types at the labial apex. Our findings will contribute to a better understanding of feeding behaviour and provide valuable insights for future taxonomic and phylogenetic research. We also summarise existing and new data on mouthpart and sensilla morphology within this diverse group.

## 2. Materials and Methods

The dry materials of particular taxa (Table 1) used in this study were obtained from the Hemiptera collection of the Natural History Museum in London. The specimens were prepared according to standard procedures for observation and imaging using a scanning electron microscope (SEM). Samples were sputter-coated with gold/palladium and examined using a SEM450 scanning electron microscope operated at 10 kV. Cross-sections of the labium in some species were made at the middle of the second segment. Samples were not sexed due to the limited number available for this study. Moreover, previous studies [14,17] indicated that the analysed mouthpart characters do not differ significantly between sexes.

The terminology for the internal structures of the maxillary and mandibular stylets follows Brożek and Herczek [29]. Sensilla classification was based on basic morphological features as described by Altner and Prillinger [47] and Zacharuck [55]. This study describes the morphology of labial tip sensilla and their corresponding sensory modalities for receiving information. The literature reveals some inconsistency in both the nomenclature and functional interpretation of sensilla, partly due to differences among taxonomic groups and partly due to challenges in distinguishing between sensilla chaetica and trichodea, trichodea and basiconica, and basiconica and peg sensilla. The sensilla were classified based on the presence or absence of pores in the sensillum wall, the sensillum length, and whether the sensillum base was embedded in a flexible or non-flexible socket. The length of the sensilla on the labial tip was measured using the scale bar in millimetres (mm) and compared with micrometres (µm) in the photos using CorelDRAW’s linear dimension tool (Table 2). An additional feature was the localisation of the sensilla. Among other objectives, this study aims to provide a more unified classification of labial tip sensilla in Cicadomorpha.

## 3. Results

### 3.1. External Structure of the Terminal Labial Segment

**Figure 1:**
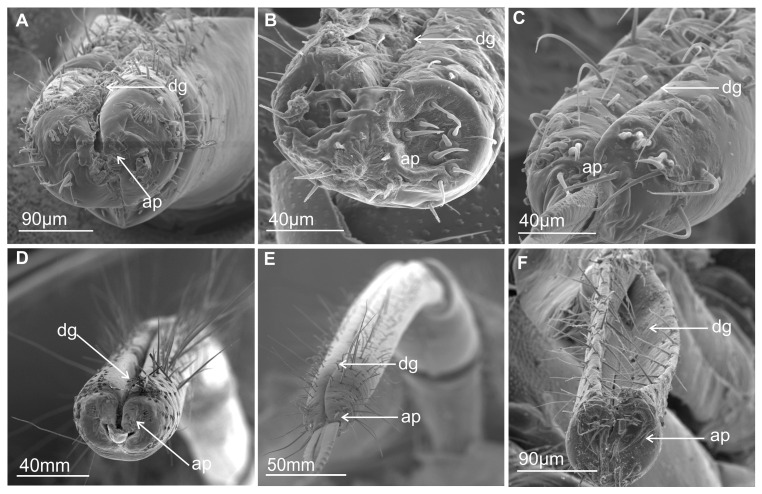
Shape of the last segment of the labium. (**A**) *Lepyronia coleoptrata* (Aphrophoridae). (**B**) *Clastpotera* sp. (Clastopteridae). (**C**) *Machaerota pandata* (Machaerotidae). (**D**) *Tettigarcta crinita* (Tettigarctidae). (**E**) *Proarna insignis* (Cicadidae). (**F**) *Eicissus decipiens* (Epipygidae). Abbreviations: dg, dorsal groove; ap, apex of the labium.

**Figure 2:**
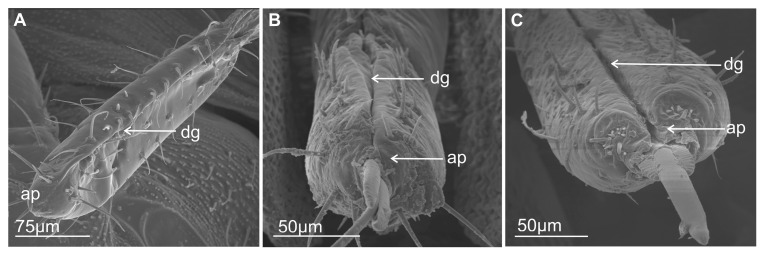
Shape of the last segment of the labium Membracoidea. (**A**) *Melizoderes* sp. (Melizoderidae). (**B**) *S. nova* (Membracidae). (**C**) *Myerslopia* sp. (Myerslopiidae). Abbreviations: dg, dorsal groove; ap, apex of the labium.

**Figure 3:**
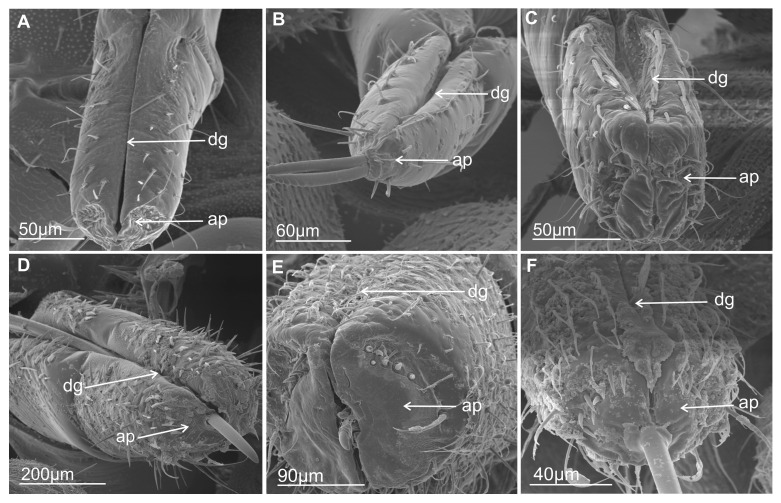
Shape of the last segment of the labium of Cicadellidae. (**A**) *D. japonicus*. (**B**) *Coloborrhis corticina*. (**C**) *Stirellus bicolor*. (**D**) *B. fulviventris*. (**E**) *C. viridis*. (**F**) *N. modulatus*. Abbreviations: dg, dorsal groove; ap, apex of the labium.

Most commonly, the edges (right and left) of the anterior side of the labium proximally are opened (Figure 1A–E) and are in contact in their distal part. The terminal segment of the entire length is evidently opened only in *Eicissus decipiens* (Epipygidae) (Figure 1F). Similarly, the last segment of the labium proximally is open in other species (Figure 2A–C and Figure 3A–F). Below their edges, a groove is formed of varying depth and position.

In some Cercopoidea: *Lepyronia coleoptrata* (Figure 1A), *Clastoptera* sp., (Figure 1B), *Machaerota pandata* (Figure 1C), in the cicadas (Figure 1D,E) and in the Membracoidea (Figure 2A–C, *Melizoderes* sp., *Stictopelta nova*, *Myerslopia* sp.), as well in *Diodontophorus japonicus* (Figure 3A) and the *Coloborrhis corticina* (Figure 3B), the groove is deep on the anterior side of the distal part of the labium, and the stylets are placed near the posterior side of the labial tip as in *Stirellus bicolor* (Figure 3C).

A shallower groove with stylets placed in the middle of the labial tip is found in *Balala fulviventris* (Figure 3D), *Ledra aurita*, *Cicadella viridis* (Figure 3E), and *Nephotettix modulatus* (Figure 3F). The apical segment is superficially grooved posteriorly in particular species but probably has no significance for the inner placement of the stylets in the labium.

Morphological studies show two types of shapes of the labial tip, either tapered as in *M. pandata* (Figure 1C), cicadas *Tettigarcta crinita* and *Proarna insignis* (Figure 1D,E), as well in representatives of the Membracoidea: *Melizoderes* sp. (Figure 2A), *S. nova* (Figure 2B), and also in the *C. corticina* (Figure 3B) or slightly rounded (bilobed) in the remainder, including *L. coleoptrata*, *Clastoptera* sp., *E. decipiens* (Figure 1A,B,F), *Myerslopia* sp. (Figure 2C), *D. japonicus* (Figure 3A), *S. bicolor* (Figure 3C), *B. fulviventris* (Figure 3D), *C. viridis* (Figure 3E), and the *N. modulatus* (Figure 3F). The periphery of the rounded tip is slightly convex but concave in its middle part (Figure 3A,C–F), whereas the tapered tip is convex and covered with a drape-like pattern of the cuticle (Figure 1C and Figure 3B).

### 3.2. External Shape and Size of the Maxillary and Mandibular Stylets

**Figure 4:**
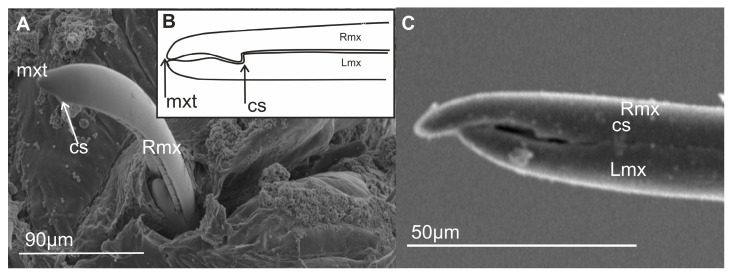
(**A**) Smooth surface of the maxillary stylets with coupling tooth (cs). (**B**) Drawing of the connections of the ends of the maxillary stylets (mxt). (**C**) Tip of the maxillary stylets in *Machaerota pandata*.

**Figure 5:**
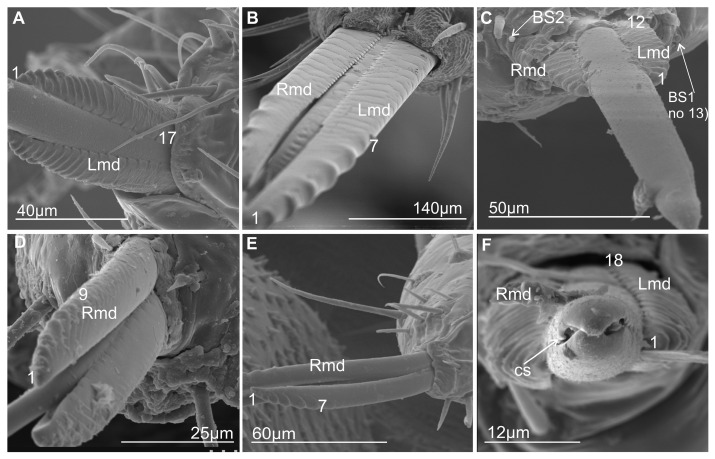
Shape of the mandibular stylets: (**A**) *M. pandata*. (**B**) *P. insignis*. (**C**) *Myerslopia* sp. (**D**) *S. nova*. (**E**) *C. corticina*. (**F**) *L. aurita*. Numbering of teeth and edges on the mandibular tip (range 1–18). BS1 and BS2, sensilla basiconica. Rmd right mandibula, Lmd left mandibula, cs coupling tooth.

The maxillary stylets (right—Rmx and left—Lmx) protrude beyond the tip of the labium in various Cicadellidae as well as in the membracid *S. nova*. These stylets are flattened dorsoventrally (Figure 4A,B). The maxillary stylets have their outer convexity complementing the inner concavity of the mandibular stylets (right—Rmd and left—Lmd) (Figure 5A–E). They are broad and flattened in their proximal third, thin in their two distal thirds, and end in a smooth, strongly pointed, and slightly curved tip (Figure 5F). The distal part of the tip is provided with a special coupling apparatus (cs) and an overlapping tooth on the right and left maxilla on each anterior and posterior surface (Figure 4A,B and Figure 5F).

The external and internal sides of the mandibles are convex and concave, respectively. They are broader in the proximal third and narrower in their two distal thirds. The tips of the mandibles have a different tooth profile. In *C. corticina* (Figure 5E), seven teeth are observed on the external side of the mandibular stylets, while some other species belonging to the various families possess a longer series of transverse ridges (about 20 ridges). A similar pattern of transverse ridges reaching the inner side of the dorsal region is found in *M. pandata* (17 ridges) (Figure 5A) and *Myerslopia* sp. (12 visible ridges; however, the mandibles were slightly protruding, so the actual number of ridges may be higher) (Figure 5C), as well as in another species (*Ledra aurita*) with a similar number of ridges (18) (Figure 5F). The ridges are most prominent at the tip of the stylet and gradually become shorter as they extend proximally until they eventually disappear near the base of each stylet. Another type of serration on the tip of the mandible consists of seven to nine larger external teeth in a proximal direction. Seven teeth were observed in *P. insignis* (Figure 5B) and *C. corticina*. Two more teeth were found in *S. nova* (Figure 5D; nine teeth). The surface of the mandibular tip varies among species, and these structures probably do not correlate with the systematic classification of species. The structure of ridges and teeth projections on the lateral fringes of the mandibular stylets yields the impression that they are flexible and may be involved in the directional movement of the maxillary stylets in the longitudinal plane of the insect.

### 3.3. Types of Labial Sensilla

Generally, insect sensilla occur in a vast variety of forms, differing conspicuously in their external morphology. Consequently, and also due to the fact that their internal structure could be only very incompletely elucidated by light microscopy, a typology has been developed that uses properties of the external morphology as criteria for differentiation.

#### 3.3.1. Mechanosensilla

**Figure 6:**
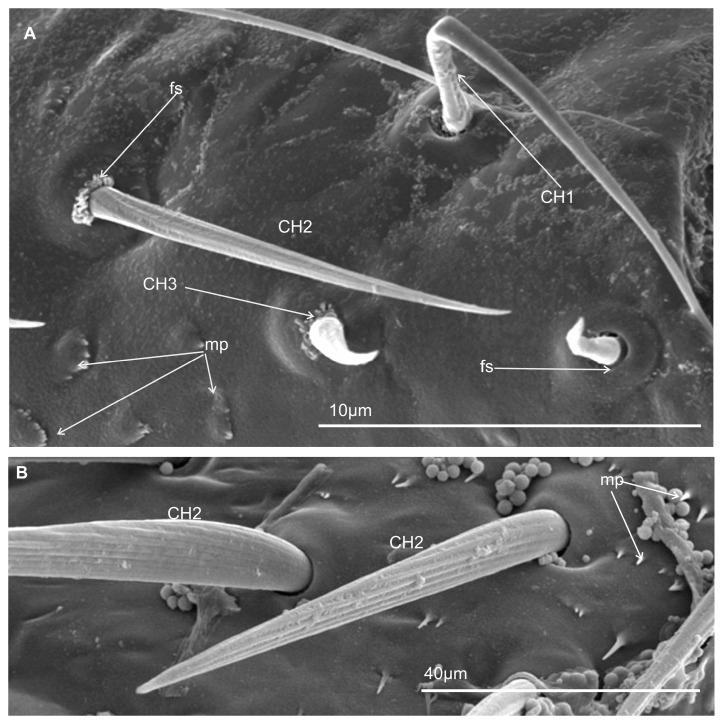
Three types of mechanosensilla of *D. japonicus*. (**A**) Length of chaetic sensilla (CH1–CH3). (**B**) Ridged surface of the chaetic sensilla. Abbreviations: fs, flexible socket; mp, cuticular processes.

**Figure 7:**
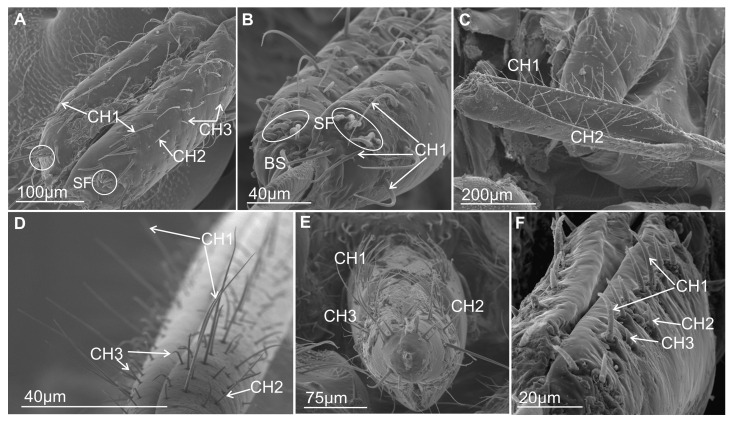
Examples of the distribution of the chaetica sensilla on the last labial segment in Cercopoidea and Cicadoidea: (**A**) *L. coleoptrata*. (**B**) *M. pandata*. (**C**) *E. decipiens*. (**D**) *P. insignis*. (**E**) *Tolania* sp. (**F**) *S. nova*. SF sensory field (circle).

This type of sensilla refers to NP–nonporous sensilla with flexible sockets (fs)—which allows for possible deformation of the sensilla. The mechanosensilla, hair-like, are the most abundant and widespread in many insects, and they are commonly called sensilla chaetica. Mechanoreceptive chaetic sensilla are straight in shape, broad at the base, and narrow at the tip. The external surface of the stem is grooved, but the pattern differs from that of the chemosensilla wall grooves. These sensilla are positioned perpendicularly or at a larger angle (about 45°) on the labial surface, making them visible as they stick out (Figure 6A,B). In leafhoppers (Figure 6A) and other species of Cicadomorpha (Figure 7A–F) on the labium of the ultimate segment, the three morphological forms of the sensilla chaetica have been observed:

- Chaetica type 1 (CH–1) (Figure 6A) are long (their length above 20 µm) and slender with shallow longitudinal grooves and a pointed tip slightly curved to the rostrum surface.
- Chaetica type 2 (CH–2) (Figure 6B) are generally of middle length (ranging from 10 to 20 µm) with longitudinal grooves.
- Chaetica type 3 (CH–3) (Figure 6A) are short (their length ranged from 4.0 to 9.0 µm) and cone-shaped with longitudinal grooves, with a curved end.

On the exposed surface in some species of the leafhoppers (e.g., *D. japonicus*, Figure 6A, as well as *B. fluviventris* and *S. bicolor*), minor pegs (mp) situated in several regular rows (probably there are no sensory structures) were visible.

#### 3.3.2. Arrangement of the Labial Mechanosensilla

All the segments of the labium have tactile hairs, but the distal segment is more strongly covered with long, recurved hairs (chaetica type 1), short hairs (chaetica type 3), which all point toward the tip, and thicker setae (chaetica type 2) (Figure 7A–F). Among the leafhoppers, the surface of the apical segment of the labium is either strongly covered by uneven chaetica sensilla (*D. japonicus*, *S. bicolor*, *B. fulviventris*, *C. viridis*, *N. modulatus*, Figure 3A,C–F) or forms a regular line as *C. corticina* (Figure 3B). In other species, chaetica sensillae (CH1, CH2, CH3) are less numerous, such as in the membracid *Tolania* sp. and *S. nova* (Figure 7E,F).

#### 3.3.3. Chemosensilla

**Figure 8:**
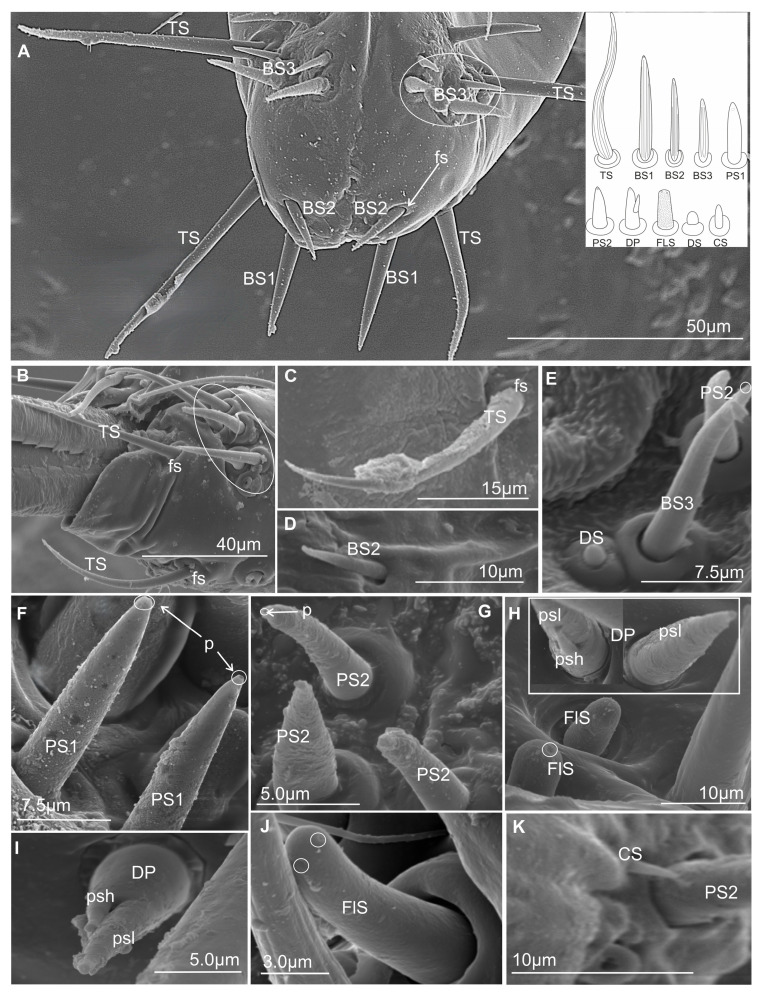
Morphological types of the labial tip sensilla. (**A**–**C**) TS sensilla trichoidea, BS1, BS2, BS3 sensilla basiconica of different length and localization; (**D**) BS2 sensillum basiconicum; (**E**) BS3 sensillum basiconicum, PS2 sensillum peg, DS sensillum dome–shaped; (**F**) PS1 sensilla peg wit visible terminal pore; (**G**) PS2 sensillum peg with visible terminal pore; (**H**,**I**) DP sensillum peg, double, long (psl) and a short (psh) peg on the basic peg; FlS sensillum finger-like; (**J**) FIS sensillum finger-like in enlargement, porus wall is visible; (**K**) CS sensillum coleoconicum and PS2 sensillum peg. P, terminal pore; fs, flexible socket.

Chemosensilla have been presented on the labial tip in three types as contact chemoreceptors, gustatory, and olfactory sensilla (Figure 8A–K).

#### 3.3.4. Contact Chemoreceptor Sensilla

**Figure 9:**
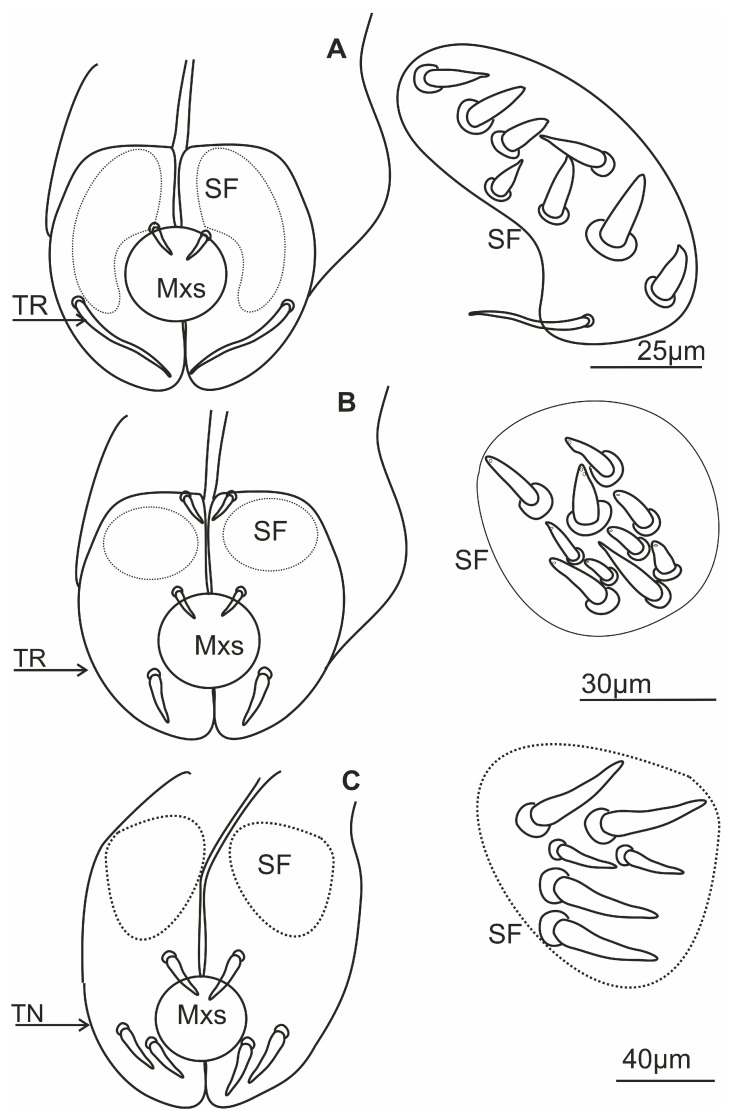
Types of the sensory field and sensilla: (**A**) Semicircular. (**B**) Rounded. (**C**) Indistinct. Abbreviations: TR, labial tip rounded; TN, labial tip narrow; SF, sensory field with sensilla; MXs, maxillary stylets.

**Figure 10:**
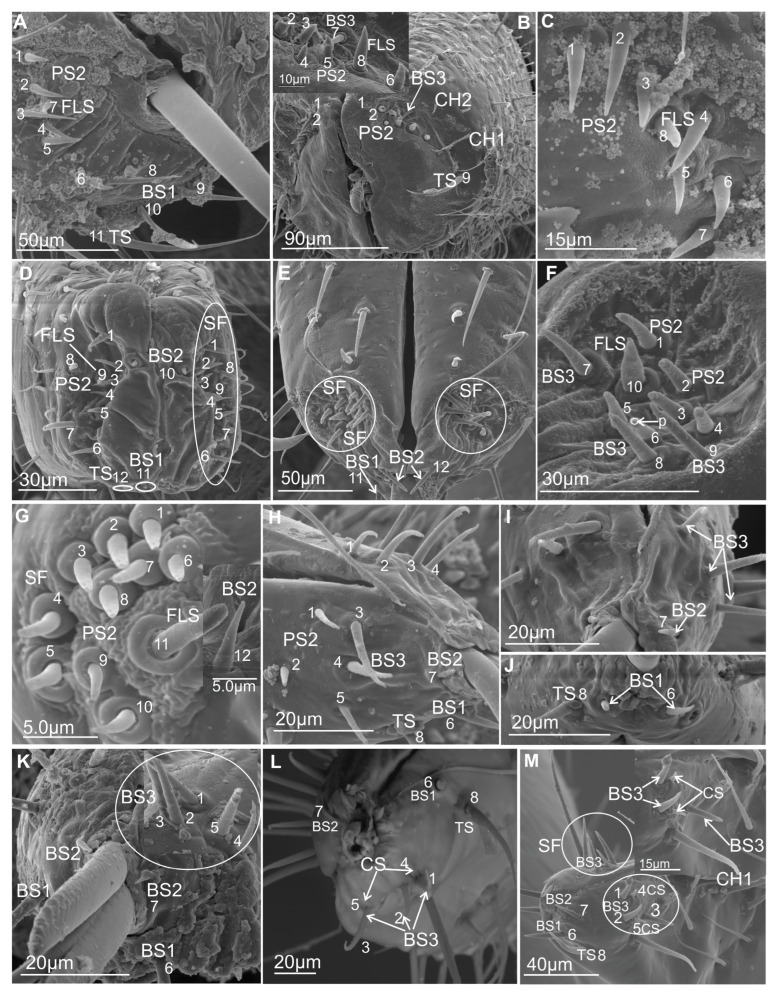
Examples of the distribution of the labial tip sensilla of Membracoidea. (**A**) *B. fulviventris*. (**B**) *C. viridis*. (**C**) *N. modulates*. (**D**) *S. bicolor*. (**E**,**F**) *D. japonicus*. (**G**) *Myerslopia* sp. (**H**–**J**) *C. corticina*. (**K**) *S. nova*. (**L**) *Aetalion* sp. (**M**) *Melizoderes* sp. Circle, sensory fields.

**Figure 11:**
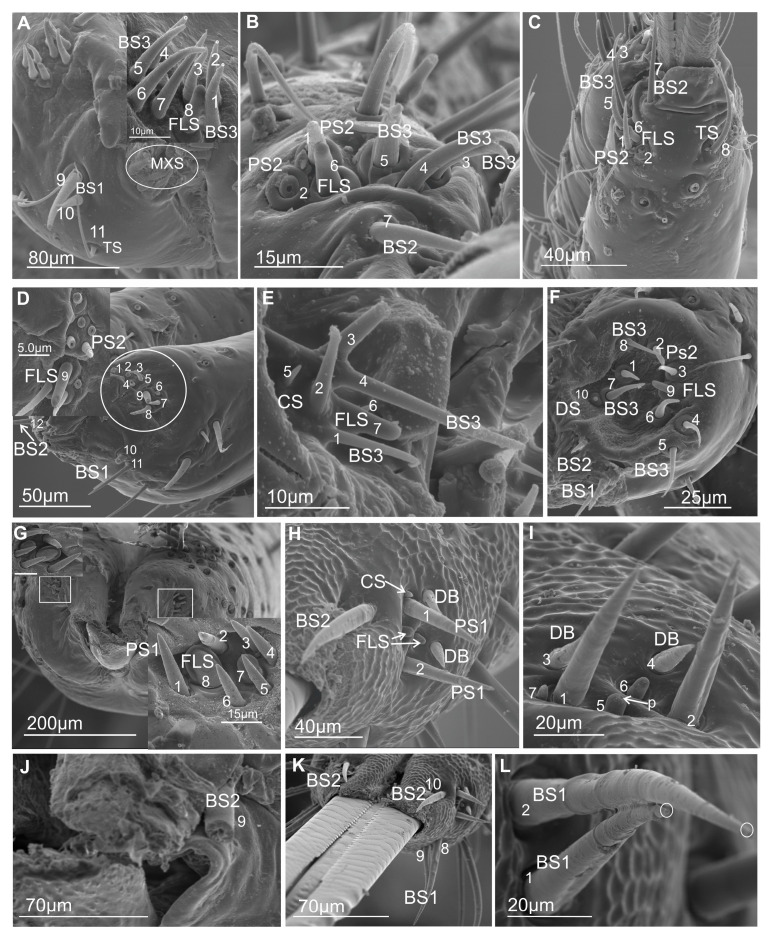
Distribution of the labial tip sensilla in Cercopoidea and Cicadoidea with sensilla in the anterior field. (**A**) *L. coleoptrata*. (**B**) *M. pandata*. (**C**) *M. pandata* (sensilla in enlargement). (**D**) *P. simulans*. (**E**) *E. decipiens*. (**F**) *Clastoptera* sp. (**G**) *T. crinita*. (**H**) *P. insignis* (lateral view). (**I**) *P. insignis* (sensilla in enlargement). Sensilla in posterior field. (**J**) *T. crinita*. (**K**,**L**) *P. insignis*.

**Figure 12:**
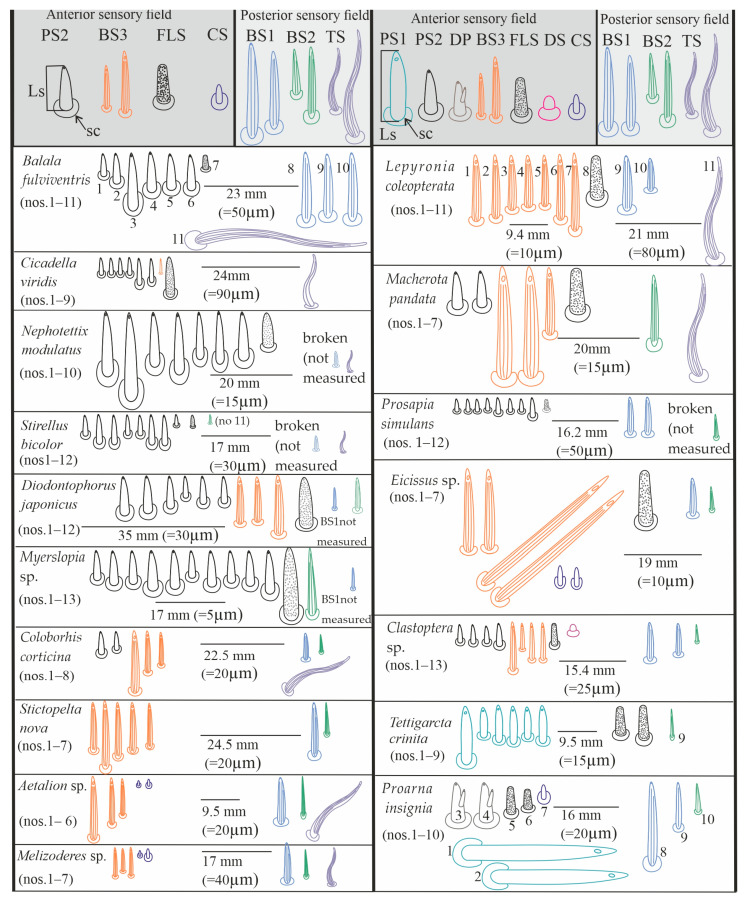
Schematic representation of the shape, size, and distribution of the labial tip sensilla, based on images from Figure 10 and Figure 11. The length (in millimetres) of the sensilla (Ls) is measured without the socket size (sc). The sensilla are labelled with numbers (nos…) as in Figure 10 and Figure 11. The scale is shown in µm and mm. Particular data on the length of the sensilla are in Table 2.

Contact chemoreceptors belong to a group of the chemo- and mechanosensilla with the TP, terminal pore, at the tip of the sensilla (also called uniporous sensilla) and sunken in flexible sockets (fs). The contact chemoreceptors, which are usually the dominant group of sensilla at the tip of the labium, are divided into two types: sensillum trichoideum (TS) and sensillum basiconicum (BS). The latter are classified into two subtypes (BS1 and BS2) based on their length (Table 2) and location (Figure 8A–D, Figure 9A–C, Figure 10A–M, Figure 11A–L, and Figure 12).

- TS–sensillum trichoidea, uniporous (Figure 8A–C) are the longest of these sensilla (ranging from 25.0 to 95 µm in length) and the slenderest of labial tip sensilla, with a curved shaft from a regular and flexible socket. It is difficult to observe the terminal pore, but the characteristics and location of the hairs suggest that they could be uniporous sensilla (chemosensilla) of the labial tip. These sensilla, located only on the middle and lower surface of the tip of the labium, are not numerous (one or two pairs were noticed).
- BS1–sensillum basiconicum uniporous (Figure 8A) are of medium length (approximately 20–45 µm) and are scattered on the ventral surface below the stylet bundles (Md and Mx). The surface of their wall is smooth; a tip is slightly tapered and possesses a terminal pore. On one side, one or two pairs of sensilla are present.
- BS2–sensillum basiconicum uniporous (Figure 8A,D) are short cones with an average length of 7–20 µm, which is always shorter than that of the sensilla basiconica (BS1) (Figure 8A). Their base is wider (2.0 µm), but the diameter measures 0.9 µm by the first quarter of the sensillum and remains this size up to the apex. These sensilla possess longitudinal ridges along their entire length or are smooth and probably have a terminal pore.

#### 3.3.5. Gustatory Sensilla

TP—terminal pore sensilla—uniporous sensilla without flexible sockets. Gustatory receptors are located among bristles, hairs, pegs, or elevations of the cuticle or beneath flat cuticular areas that have a single terminal pore [47]. According to morphological, external features, these sensilla should be regarded as chemosensilla (gustatory ones). The three types of sensilla have been distinguished as gustatory (BS3, PS1, PS2, DP).

- BS3–sensillum basiconicum uniporous (Figure 8E) is slim and of middle length (6–20 µm), with slightly longitudinal ridges along its whole length and a terminal pore. In most species, the average length of the sensilla is around 20 µm, while the shortest are found in Cicadella (6 µm), in contrast to the longest, which are found only in *Aetalion* sp. (24 µm) (Table 2). These sensilla are longer than the sensillum peg and, in several numbers, are present on the anterion sensory fields.
- PS–sensillum peg uniporous (Figure 8E–G) are uniporous cones with slightly rounded tips. The surface of these sensilla is smooth along their entire length, and the terminal pore is clearly visible (Figure 8F,G). The base of the sensillum is wider than the blunted tip. Based on their different lengths, two subtypes are recognised: PS1 and PS2. PS1 is longer, at around 20.0 µm (Figure 8F), and the longest are observed in (*P. insignis*) (Table 2). PS2 has a length ranging from 2.0 to 19.0 µm. Sensilla are arranged in the sensory field, and there are numerous sensilla in one field, from 1 to 10.
- DP–sensillum peg, double uniporous (Figure 8H,I), has a base of the cone with a long (psl) and a short (psh) peg. Probably both pegs of this sensillum possess the terminal pore. This sensillum was found only in one species (*P. insignis*).

#### 3.3.6. Olfactory Sensilla

Sensilla include wall-pore sensilla with an inflexible basal socket [47], multiporous sensilla, and thin-walled sensilla [55]. They all have a relatively large number of pores in common. These sensilla project from the body cuticle surface, but some are in sockets or pits, both deep and shallow. They vary greatly in form and size. In representatives of Cicadomorpha families, one type of sensory organ has been recognised.

- FlS–sensillum finger-like (Figure 8H,J) is a thick cone sunken in a shallow socket. They can be compared to short sensilla pegs but with a strong, rounded tip (Figure 8H). One or two pairs of sensilla are present in some species. Their surface is covered with undulated grooves and pitted. Terminally, the rounded tip of these sensilla possesses one or two larger pores than wall multipores (Figure 8H,J). Usually, these sensilla ranged from 6 to 10 µm in length; however, in one species (*Cicadella viridis*), an excessive length of 36.7 µm was observed (Table 2).

#### 3.3.7. Thermo-Hygrosensilla

Sensilla are non-porous, thin-walled, and embedded in an inflexible socket [47]. Some are protruding from the surface of the cuticle or are localised in deeper pits. They vary greatly in form and size. In representatives of families of Cicadomorpha, two shapes of these sensilla have been recognised.

- DS–sensillum dome-shaped (Figure 8E) is low with a rounded tip. This type of sensillum is situated on the sensory field near basiconica sensilla. This sensillum was found only in species of Clastoptera.
- CS–sensillum coleoconicum (Figure 8K), the thin pegs are inserted in a shallow cavity on the sensory field. This type of sensillum is very short (about 3 to 5 µm) and placed near the sensillum basiconicum.

### 3.4. Distribution of the Labial Tip Sensilla Membracoidea

The distal tip of the labial segment bears different sensilla (mechanosensilla, chemosensilla, and thermo-hygrosensilla), which are often grouped in the upper (anterior) sensory fields and are symmetrically located around the labial groove (one on the right and one on the left). In the present morphological description, the arrangement and number of sensilla refer to the field on one side of the labial apex (Figure 9A–C and Figure 10A–M). Some sensilla are arranged above or more laterally than the stylet exit and below it (Figure 9A–C). These sensilla are usually located singly or in pairs and form a posterior field. The margin of the labial tip is often equipped with mechanosensilla, which probably detect the texture of the plant’s surfaces (Figure 10G,I–K).

Near the anterior surface of the labial tip above the stylets is a sensory field (SF) (Figure 9A–C and Figure 10A–M). In *B. fulviventris* (Figure 10A), *C. viridis* (Figure 10B), *N. modulatus* (Figure 10C), and *S. bicolor* (Figure 10D), the anterior field is a semicircular cavity. In other leafhopper species, e.g., *D. japonicus* (Figure 10E,F) or *Myerslopia* sp. (Figure 2C and Figure 10G), the sensory field resembles a round cavity. In *C. corticina* (Figure 10H–J), *S. nova* (Figure 10K), *Aetalion* sp. (Figure 10L), and *Melizoderes* sp. (Figure 10M), the sensory field is narrowed. In the anterior field of the labial apex, several sensilla form a distinct set. These include sensilla: basiconica (BS3), peg (PS2 and PS1), double peg (DB), dome-shaped, coeloconic, and finger-like. In the middle part of the labial tip, close to the stylets’ protrusion, there are usually sensilla basiconica (BS2), which probably have a mechanical or contact-chemoreception function. In some species, this sensillum is absent. Below the stylets, the posterior sensory field comprises one trichoideum sensillum (TS) and one or three basiconica sensilla (BS1), which act as contact chemoreceptors. The numbers and arrangement of the sensilla for a particular species are detailed and presented below.

In *B. fulviventris* (Cicadellidae: Hylicinae) (Figure 10A), there are six uniporous peg sensilla (PS2, nos. 1–6) and one finger-like sensillum (FLS, no. 7), which is partially hidden between PS2. Below the stylets are three sensilla basiconica (BS1, nos. 8–10) and one sensillum trichoideum (TS, no. 11). The sensillum basiconicum (BS2) was not observed. In *C. viridis* (Cicadellinae) (Figure 10B), six peg sensilla (PS2, nos. 1–6), one sensillum basiconicum (BS3, no. 7), and a sensillum finger-like (FLS, no. 8) are observed. Only the sensillum trichoideum (TS, no. 9), a contact chemoreceptor, is observed in the middle position of the stylets. Below the stylets, no sensilla were observed. In *N. modulatus* (Deltocephalinae) (Figure 10C), seven sensilla pegs (PS2, nos. 1–7) were present, whereas an additional peg sensillum (PS2, no. 8) was present in *S. bicolor* (Deltocephalinae) (Figure 10D). Both species *N. modulatus* and *S. bicolor* have one sensillum finger-like (FLS, nos. 8 and 9 respectively). Furthermore, in *S. bicolor*, one sensillum basiconicum (BS2, no. 10) is above the stylet, whereas in *N. modulatus*, it is absent (Figure 3F). In both species, the basiconic (BS1) and trichoid (TS) sensilla are present (in Figure 3F and Figure 10D, the socket and basic part of the sensilla were documented, because these were broken). In *D. japonicus* (Cicadellidae: Errhomeninae) (Figure 10E,F), the anterior field comprises six sensilla peg (PS2, nos. 1–6), three sensilla basiconica (BS3, nos. 7–9), and a finger-like sensillum (FLS, no. 10). Also, sensilla basiconica (BS2, no. 12) is present above, and BS1 (no 11, visible only basic part) is present below the stylets (Figure 10E). In *Myerslopia* sp. (Myerslopiidae: Myerslopiinae) (Figure 10G), there are ten robust, uniporous sensilla pegs (PS2, nos. 1–10) and multiporous sensillum finger-like (FLS, no. 11) and one sensillum basiconicum (BS2, no. 12). The BS1 is probably broken (Figure 5C). In *C. corticina* (Ulopidae: Ulopinae) (Figure 10H–J), there is a smaller number of sensilla, two sensilla pegs (PS2, nos. 1, 2) and three sensilla basiconica (BS3, nos. 3–5). However, the sensillum finger-like was not observed. The sensillum basiconicum (BS1, no 6) is present similarly, as well as sensilla basiconica (BS2, no. 7) and trichodea (TS, no. 8). In *S. nova* (Membracidae: Darninae) (Figure 10K) and *Tolania* sp. (Nioamiinae) (Figure 7F), the five sensilla basiconica (SB3, nos. 1–5) are present; however, sensillum no. 5 is morphologically similar to a sensillum finger-like. The sensilla basiconica (BS1, no. 6) (BS2, no. 7) are present; however, trichoid sensillum (TS) was not found. In *Aetalion* sp. (Aetalionidae: Aetalioninae) (Figure 10L) and in *Melizoderes* sp. (Melizoderidae: Melizoderinae) (Figure 10M), the anterior sensory fields possess three sensilla basiconica (BS3, nos. 1–3) and two sensilla coeloconica (CS, nos. 4–5). In both taxa, the sensillum basiconicum (BS1, no. 6) is similarly located, as well as sensilla basiconica (BS2, no. 7) and trichodea (TS, no. 8).

The overall membracoid pattern of the labial tip sensilla, according to the samples, concerns a similarity in representatives of the Aetalionidae, Membracidae, and Melizoderidae. On the other hand, a similarity was found between Myerslopiidae and Cicadellidae, except for *C. corticina*.

### 3.5. Distribution of the Labial Tip Sensilla of Cercopoidea and Cicadoidea

The organisation and the types of labial sensilla present in the studied families (Aphrophoridae, Machaerotidae, Epipygidae, Cercopidae, Clastopteridae, Tettigarctidae, and Cicadidae) at the tip look evidently diversified (Figure 11A–I). The number of sensilla ranges from 8 to 10 on one side of the anterior sensory field. Sensilla are clearly arranged in a rounded cavity of the field in most tested species except *P. insignis* (Cicadidae) (Figure 11H,I). In *L. coleoptrata* (Aphrophoridae) (Figure 11A), seven sensilla basiconica (BS3, nos. 1–7) and one sensillum finger-like (no. 8) form two groups (4BS3 + (3BS3 + 1 FLS)). Below the stylets, there is a pair of sensilla basiconica (BS1, nos. 9 and 10) and one long, smooth sensillum trichoideum (TS, no. 11). The sensillum basiconicum (BS2) was absent. In *M. pandata* (Macherotidae) (Figure 11B,C), two sensilla pegs (PS2, nos. 1 and 2), three sensilla basiconica (BS3, no. 3–5), and one sensillum finger-like (FLS, no. 6) form two groups (3BS3 + (2PS2 + 1FLS)). One sensillum (BS2, no. 7) and a sensillum trichoideum (TS, no. 8) were observed. In *P. simulans* (Cercopidae) (Figure 11D), the sensilla peg (PS2, nos. 1–8) and the sensillum finger-like (FLS, no. 9) form two groups (5BS3 (3BS3 + 1FLS)). Furthermore, two sensillum basiconicum (BS1, nos. 10 and 11) and one sensilla basiconica (BS2, no. 12) were evident. In other taxa, such as *Eicissus* sp. (Epipygidae) (Figure 11E), four sensilla basiconica (BS3, no. 1–4), two sensilla coeloconica (CS, no. 5 and 6), and one sensillum finger-like (FLS, No. 7) were found. The posterior field with sensilla was not presented in the figures; however, in this study, such sensilla, BS1 and BS2 in one pair, were observed in other SEM figures. In *Clastoptera* sp. (Clastopteridae) (Figure 11F), there are four sensilla peg (PS2, no. 1–4) and four sensilla basiconica (BS3, No. 5–8), as well as a visible sensillum finger-like (FLS, no. 9). Among the more specific types of sensillum, a dome-shaped multiporous sensillum (DS, no. 10) is present. No changes were observed in the distribution of sensilla basiconica (BS1, nos. 11 and 12 and BS2 no. 13) below and above the stylets of *Clastoptera* sp. In *T. crinita* (Figure 11G) (Tettigartidae), six robust sensilla pegs (PS1, nos. 1–6) and two sensilla finger-like (FLS, nos. 7–8) were observed. In contrast, the sensilla in *P. insignia* (Cicadidae) varied more in shape (Figure 11H,I). In the concave anterior field, the following were present: two sensilla pegs (PS1, nos. 1, 2); two double sensilla basiconica (DB, nos. 3, 4); two sensilla finger-like (FLS, nos. 5, 6); and one sensillum coeloconica (CS, no. 7). In *P. insignis,* two sensilla basiconica (BS1, 8, 9) were found under the stylets (Figure 11K,L). Both species have sensilla basiconica (BS2): in *T. crinita* (Figure 10J, BS2, no. 9) and in *P. insignia* (Figure 11K, BS2, no. 10).

### 3.6. Scheme of the Connecting System of the Mandible and Maxillae

**Figure 13:**
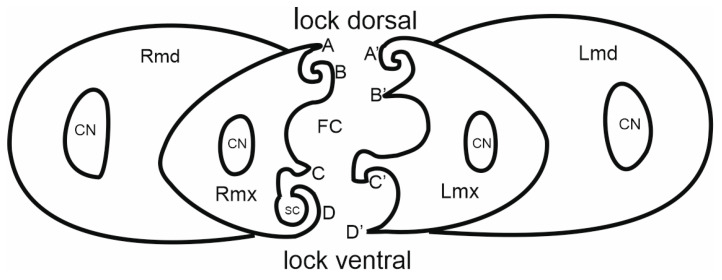
Diagram of a cross-section across the subapical rostral segment of Cicadomorpha: maxillae with two locks. Abbreviations: A, straight upper right process of lock; the dorsal A’, hooked upper left process of the dorsal lock; B, hooked lower right process of the dorsal lock; B’, straight lower left process of the dorsal lock; C, straight upper right process of the ventral lock; C’, hooked upper left process of the ventral lock; D, straight lower right process of the ventral lock; D’, hooked lower left process of the ventral lock; LMx, left maxilla; RMx, right maxilla; LMd, left mandible; RMd, right mandible; CN, nervous canal; SC, salivary canal; FC, food canal.

Internally, the maxillary stylets are interlocked by a system of ridges and grooves. In the mouthparts of cicadomorphans, the connection between the right (RMx) and left (LMx) maxillae consists of two (dorsal and ventral) locking systems. The maxillae are joined over most of their length by a special interlocking system that brings together variously shaped ridges and grooves (Figure 13). The ridges in cross-section appear as processes and therefore this term is used here. The terms used to describe these processes derive from their shape and position (Figure 13); two processes in the locks of the maxilla (one above the other) are called the upper (A’, C’) and the lower (B’, D’) processes. Four processes form the dorsal and ventral lock of the maxilla. On the dorsal lock of the right maxilla, an upper straight process (A) and lower hooked process (B) are present. On the left maxilla, the arrangement of processes is opposite: an upper hooked (A’) and a lower straight process (B’). On the ventral lock of the right maxilla, a straight (C) and hooked (D) process is present. On the left maxilla, a hooked (C’) and straight (D’) process is present. The left hooked (C’) process is joined to the straight process (C) and hooked (D) on the right maxillae. The hooked (C’) process closes the salivary canal (Figure 12).

#### 3.6.1. Maxillae and Mandibles in Cross-Section

**Figure 14:**
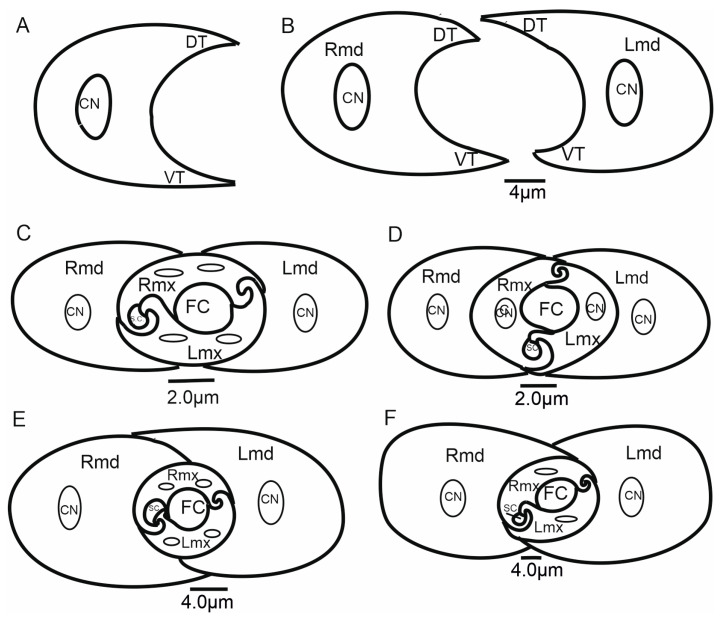
Cross-sectional thoughts of the stylet bundle of Cicadellidae and Membracidae: (**A**) mandible symmetrical with a narrow tip DT (dorsal tip) and VT (ventral tip). (**B**) Mandibles are asymmetrical, with the dorsal tip and ventral having different lengths. (**C**) *Idiocerus stigmaticalis*. (**D**) *P. nodosus*. (**E**) *S. nova*. (**F**) *Neotituria kongosana*.

In cross-section, mandibles appear to be crescent-like and wider medially (Figure 14A,B). The internal and external sides of the mandibles are convex and concave, respectively, and are able to slide along the walls of the maxillae. On the left and right mandibles, dorsal (DT) and ventral (VT) mandibular tips are equally long, pointed, and usually symmetrical (Figure 14A) or asymmetrical (Figure 14B).

**Figure 15:**
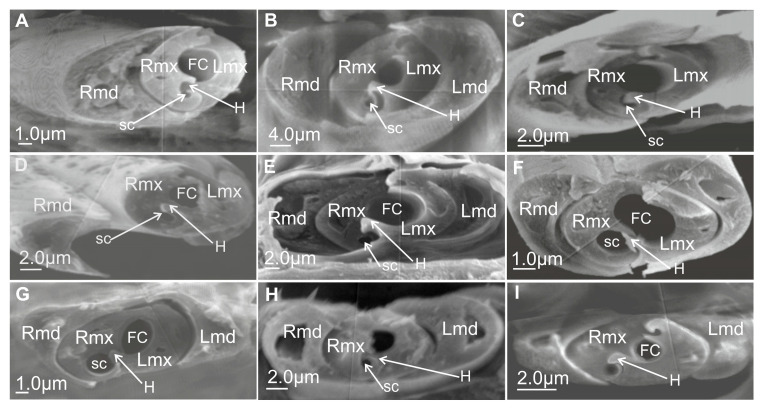
A cross-section of stylet bundles in different species of Cicadellidae and Membracidae. (**A**) *U. reticulata*. (**B**) *I. lanio*. (**C**) *M. fuscinervis*. (**D**) *S. agrestis*. (**E**) *E. gratiosa*. (**F**) *K. bipunctata*. (**G**) *E. vitatta*. (**H**) *S. sordidus*. (**I**) *D. stylata*. Abbreviations: Lmx, left maxilla; Rmx, right maxilla; Lmd, left mandible; Rmd, right mandible; SC, salivary canal; FC, food canal; H, process hooked.

**Figure 16:**
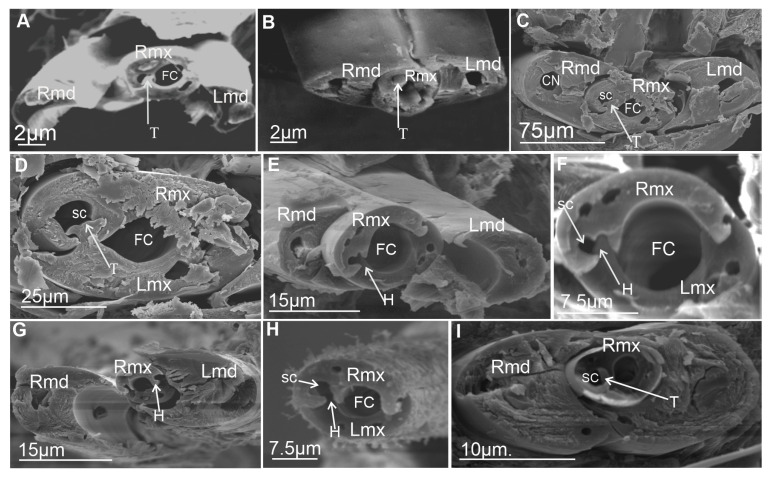
Cross-section by stylet bundles in distal part of labium in Cercopoidea, Cicadoidea, and Membracoidea: (**A**) *A. costalis*. (**B**) *C. podolica*. (**C**,**D**) *T. crinita*. (**E**,**F**) *Myerslopia* sp. (**G**,**H**) *Aetalion* sp. (**I**) *Tolania* sp. Abbreviations: Rmd, right mandibula; Lmd, left mandibula; Rmx, right maxilla; Lmx, left maxilla; SC, salivary canal; FC, food canal; T, T-shaped processes; H, hooked processes.

The symmetrical shape of the mandibles is dominant in most of the leafhoppers (Figure 14C,D and Figure 15A,C–F,H,I) and other species of different families (Figure 16A–G). Small differences in the shape of mandibles in particular species were observed (*Kyboasca bipunctata,* Figure 15F, *Eupteryx vitatta,* Figure 15G). Asymmetrical mandibles, where the shape of the right mandible is the reverse of the left, are characteristic of Ledrinae (*Neotituria kongosana* Figure 14F), Iassinae (*Iassus lanio*, Figure 15B), Typhlocybinae (*E. vitata,* Figure 15G), and the membracid (*S. nova,* Figure 14E, *Tolania* sp., Figure 16I). Depending on their size, the mandibles can entirely surround the maxillae, with the dorsal and ventral mandibles in contact with *Ulopa reticulata* (Figure 15A), *I. lanio* (Figure 15B), *Macropsis fuscinervis* (Figure 15C), *Strogglyocephalus agrestis* (Figure 15D), *Edwardsiana gratiosa* (Figure 15E), *Aetalion* sp. (Figure 16G,H), and *Tolania* sp. (Figure 16I). Where this is not the case, the tips of the left and right mandibles do not touch dorsally and ventrally: *K. bipunctata* (Figure 15F), *Streptanus sordidus* (15H), *Doratura stylata* (Figure 15I); *Aphrophora costalis* (Figure 16A), *Cicadetta podolica* (Figure 16B), *T. crinita* (Figure 16C,D), and *Myerslopia* sp. (Figure 16E,F).

In cross-section, the joint connecting the maxillae (right–Rmx and left–Lmx) in Cicadellidae and other representatives of Cicadomorpha is an oval assemblage (Figure 15A–I). Internally, the maxillae are not bilaterally symmetrical. The following three positions of the maxillae are apparent.

1. Diagonal: The dorsal and ventral locks are turned to the left in relation to a perpendicular line (Figure 14B,F). Observed only in Ledrinae and Iassinae in the proximal part of the stylets.
2. Perpendicular: With the dorsal and ventral locks in a perpendicular line, generally in the proximal part of the stylets. Present in various cicadellid subfamilies (Ulopinae, Macropsinae, Aphrodinae, Typhlocybinae, Cicadellinae, and Deltocephalinae, Figure 14D and Figure 15A–I).
3. Horizontal: When the dorsal and ventral locks lie inside the internal concavity of the mandibles (Figure 14C,E). Present in representatives of Cercopidae, Cicadidae, Tettigarctidae, Myerslopiidae, Aetalionidae, and Membracidae (Figure 16A–I).

#### 3.6.2. Salivary and Food Canals

Each of the maxillary stylets has a wide concavity on its inner surface. From the inside, the maxillae form two canals: salivary (SC) and food (FC) (Figure 13 and Figure 14C–F).

In all studied leafhopper species, the food canal is formed by both maxillae in equal proportions, and it is placed centrally in the subdorsal portion of the maxillae. It is usually circular in cross-section due to its concave inner wall.

The salivary canal is suboval and placed only in the right portion of the maxillary stylets in a ventral position (Figure 14C–F). In all Cicadellidae and Membracidae (except *Tolania* sp., Figure 16I) examined, a hooked process (C’) closes the left maxilla. The hooked process (C’) (Figure 14C, Figure 15A–I and Figure 16F–H) was also found in representatives of other families, whereas in Cicadidae, Cercopidae, and Aphrophoridae, a T-shaped process is found (Figure 16A–D).

## 4. Discussion

Despite extensive research on the mouthparts of Hemiptera, relatively little attention has been given to Cicadomorpha, particularly leafhoppers. While existing studies based on a limited number of species suggest that leafhopper mouthparts are generally similar in structure, with variations largely restricted to proportions and fine details such as the number and arrangement of sensilla on the labium and the surface sculpturing of the stylets, comprehensive comparative analyses across a broader range of species are lacking. Such studies are essential in order to determine the extent of morphological variation among major lineages and to assess its relevance with regard to systematics, evolutionary relationships, and feeding adaptations.

### 4.1. Labium-Shaped and Stylet Bundles

The basal morphological features of the labium in Cercopoidea and Cicadoidea are generally similar to those in the Membracoidea, many families of leafhoppers and treehoppers. In most membracoid species that have been studied, the edges of the labial groove are open proximally (usually in the first segment) and meet in the second and third segments. However, *E. decipiens* (Epipygidae) has a clearly open terminal segment along its entire length. The shape of these edges determines the depth and position of the groove formed. A deep groove in the distal part of the labium with stylets placed near the posterior side of the labial tip has been observed in Cercopoidea (*L. coleoptrata*, *Clastoptera* sp., *P. simulans,* and *M. pandata*); Cicadoidea (*T. crinita* and *P. insignis*); and Membracoidea (*Melizoderes* sp., *S. nova,* and *Myerslopia* sp.). In Cicadellidae, however, the groove is shallow, and the stylets are positioned in the middle of the labial tip, as seen in *S. bicolor*, *B. fulviventris*, *L. aurita*, *C. viridis,* and *N. modulatus*. The apical segment is superficially grooved posteriorly in certain species, but this is probably not significant in terms of the inner placement of the stylets in the labium.

Morphological analyses also reveal two types of labial tip shape: tapered and rounded. The latter has a slightly convex periphery and a concave middle part and is found in most Cercopoidea and Membracoidea. In contrast, the tapered tip is convex and covered with a drape-like pattern of cuticle in both studied cicadas and in some of the Membracoidea (*Melizoderes* sp., *S. nova,* and also in the *C. corticina*) and Cercopoidea (*M. pandata*). Notably, the shape of the maxillary and serrated mandibular stylets can differ between species. Wang et al. [54] described the mouthparts of *Philagra albinotata* Uhler (Hemiptera: Cercopoidea: Aphrophoridae), highlighting a distinctive characteristic of this species. According to these authors, *P. albinotata* has a tooth between the maxillary stylets at their tips—a feature not observed in other Cicadomorpha and Fulgoromorpha species. Carlson et al. [15] studied the mouthparts of 11 species belonging to six subfamilies of membracids, analysing their structural intricacies. These authors described the maxillary stylets as being smooth, curved, and acute at the apex and relatively uniform across the six genera studied. The current study revealed a distinctive coupling apparatus in Membracoidea (*Myerslopia* sp. and *L. aurita*; Figure 5C,F), comprising overlapping teeth on the anterior and posterior surfaces of the right and left maxillae. The maxillary structures in *P. albinotata* and the aforementioned Membracoidea species appear to be very similar. Also, a similar shape of the apex of maxillary stylets was visible in *M. pandata* (Cercopoidea). A deeper morphological analysis of the maxillary stylets in other species could confirm this. Furthermore, Wang et al. [54] found that the mandibular stylets of *P. albinotata* contained numerous small barbs. The distal tips of the mandibular stylets were toothed and resembled a series of barbed ridges found in cicadellids such as *Macrosteles fascifrons* [19], *H. coagulata* [13], and *Psammotettix alienus*—in publication [24] as *P. striatus*. In six subfamilies of membracids, only a row of recurved barbs in the mandibular stylets was identified, similar to those previously described in other leafhopper species [15]. In contrast, our study reveals changes to the profile of the mandibular tips, with a shift from seven to almost twenty teeth or ridges across different species and taxonomic groups. Furthermore, different patterns emerge: regular, long transverse ridges in *M. pandata*, *Myerslopia* sp., and *L. aurita*, as opposed to six or seven larger external teeth in a proximal direction and numerous smaller dorsal inner teeth in *P. insignia*, *S. nova,* and *C. corticina*. The range of serration of mandibular stylets can vary within a family. A study by Tavella and Arzone [16] showed that the mandibular stylets of cicadellid species differ in shape and number of serrations. *Z. pullula* and *H. vitis* had a similar number (9–11) of prominent dentations, whereas *G. fennahi* had around 20 hardly projecting dentations. The surface of the mandibular tip is varied in many species, and these structures probably do not correlate with systematic groups of the species. Variation in the mandibular stylet morphology, particularly in the number and shape of the small barbs or teeth, may represent adaptations to different host plant tissues or feeding sites. This aligns with previous suggestions that mandibular modifications can reflect feeding niche specialisation [31]. The structure of ridges and teeth projections on the lateral fringes of the mandibular stylets yields the impression that they are flexible and may be involved in the directional movement of the maxillary stylets in the longitudinal plane of the insect. Apparently, stylet movement in the transverse direction in related cicadellid species is accomplished by retraction of one of the mandibulary or maxillary stylets so that pressure on the leading end of the other stylet causes a deflection in the direction of the incurved tip [13,25]. Further detailed studies of the microstructure of stylet teeth, based on more abundant material, are crucial for analysing the evolutionary and trophic relationships across taxa in Cicadomorpha. As this study was based on only a few species, it has merely highlighted further research questions and hypotheses to be tested in this area.

Are specific types of mandibular serrations in species correlated with feeding on particular host plants? Has the apex of the maxillae been subject to evolutionary pressures leading to morphological diversification?

### 4.2. Labial Tip Sensilla

The gross morphology of the labial tip sensilla in Cicadomorpha is similar to that reported in planthoppers and other hemipteran groups [20,53]. Their location and types are consistent with their function in recognising the plant surface. In relation to their host plant range, they may therefore carry some taxonomic/phylogenetic information that has not yet been investigated in this context.

Information about volatiles emanating from plant surfaces and how these volatiles interact with insect cuticles is obtained through the exploration of plant surfaces by heteropteran bugs using their antennal flagellum [49,53]. This suggests that this process involves contact-chemoreceptive sensilla with a gustatory function. However, these sensilla may also be associated with olfactory function [55]. In contrast, leafhoppers and planthoppers probably do not use antennation when exploring plant surfaces [53]. Their antennae are small, and the chemosensilla are located on the basal part of the flagellum in leafhoppers and in the pedicel in planthoppers, neither of which directly touches the plant surface. Consequently, it is likely that exploring the plant surface has become the function of the apex of the labium in these insects. They dab the plant surface with contact-chemoreceptive sensilla or smaller peg/basiconic sensilla with a gustatory function [53,56,57]. In Fulgoromorpha, some sensilla on the labial tip have been shown to have an olfactory function [50]. This appears to be an example of exaptation, i.e., the secondary acquisition of a new function by an existing structure [50].

According to our current data, the labial tip of the Cicadomorpha species studied is equipped with a varied set of sensilla. These include various types of olfactory and gustatory sensilla, as well as thermo-hygroreceptive sensilla and standard mechanosensilla. There are also dual-function contact chemoreceptors. Their function can be inferred from their detailed morphology and localisation. The most important chemosensory sensilla are the gustatory peg/short basiconic sensilla and one or two finger-like olfactory sensilla located on the labial tip. The latter type has been found in several species. Short or longer basiconic sensilla, and more rarely trichoid sensilla, are present in one or more pairs and belong to the contact-chemoreceptor system. Coeloconic and dome-shaped sensilla with thermo-hygroreceptive function are less numerous and difficult to observe. These sensilla are very short and are often hidden near the base of the other sensilla. The mechanosensilla that cover the surface of all the last labial segments in our study are classified as chaetic sensilla in three forms (CH1–long; CH2–middle length; and CH3–short) based on the distinctive morphological characteristics proposed by Althner and Prillinger [47] and Shield [58].

Due to different classifications of sensilla, analysis of the sensilla at different locations on the labium and labrum, and detailed morphology of sensilla, it is difficult to make one-to-one comparisons of the labial sensilla of the species of Cicadomorpha studied here with previous authors’ data. Therefore, the sensilla in this discussion analysis are cited under different names (symbols) and numbers; however, we try to assign their base morphologies and functions.

This study showed that both *T. crinita* (Tettigartidae) and *P. insignia* (Cicadidae) possess a complex set of sensilla, including gustatory (sensilla peg), olfactory (sensilla finger-like), and thermo-hygroreceptive (sensilla coeloconica) structures. Notably, *P. insignia* displayed double basiconic and coeloconic sensilla, which are absent in *T. crinita*. These structures may represent potential autapomorphies of the Cicadidae family and may indicate a higher degree of specialisation in plant perception. In both species, a single basiconic sensillum (BS2), which probably functions as a mechanoreceptor or contact chemoreceptor, is located laterally to the stylets. *P. insignis* has two additional basiconic sensilla (BS1), which are located beneath the stylets and identified as contact chemoreceptors.

Hao et al. [52] identified several basiconic sensilla (Sb4) and a single finger-like sensillum with a smooth surface in nymphs of *Meimuna mongolica* (Distant) (Cicadidae). In adults, the basiconic sensilla resembled those found in nymphs, but the finger-like sensillum was absent. This study revealed ontogenetic differences, with sensilla present in nymphs regressing in adults, suggesting functional variability depending on developmental stage. In contrast, our study confirms the presence of finger-like sensilla in adult specimens of both Cicadidae and Tettigartidae. Interestingly, the finger-like sensillum found in the *M. mongolica* nymph is morphologically similar to those found in the adult forms studied here. Furthermore, the basiconic sensilla (Sb4) correspond to the gustatory sensilla peg (PS1) identified in our analysis.

In *L. coleoptrata* (Aphrophoridae), *M. pandata* (Macherotidae), *E. decipiens* (Epipygidae), *P. simulans* (Cercopidae), and *Clastoptera* sp. (Clastopteridae), the anterior sensory field is filled by gustative sensilla basiconica (BS3) and sensilla peg (PS2) that are numerous (three to seven). Also, one of the finger-like sensilla is found. Among the more specific types of sensilla, the dome-shaped multiporous sensillum (DS) is present only in Clastopteridae. The only slight difference is observed in the presence or lack of the sensilla basiconica BS1 and BS2.

A comparative analysis of the labial tip sensilla in two species of the Aphrophoridae family—*Philagra albinotata* and *L. coleoptrata*—revealed only minor morphological differences. However, some variation in sensillum types and arrangement suggests potential differences in sensory specialisation.

According to Wang et al. [54], *P. albinotata* has ten peg sensilla arranged in two groups, as well as a single large peg sensillum in the anterior sensory field. Four trichoid sensilla are present in the posterior field. These structures are presumed to function primarily as chemoreceptors and mechanoreceptors that are potentially involved in host plant detection and assessment. In contrast, our observations of *L. coleoptrata* revealed a more diverse array of sensilla on the labial tip. Specifically, the anterior field contains seven basiconic sensilla of the BS3 type (uniporous and gustatory), arranged in two distinct groups: one with four BS3 sensilla, and one with three BS3 sensilla accompanied by a finger-like sensillum (FLS). The FLS is a porous structure presumed to have an olfactory function. Above and near the stylets, a single basiconic sensillum (BS2) is present. Below the stylets, there is a pair of contact chemoreceptors (BS1) and one long, smooth trichoid sensillum (TS), which is likely to serve a contact-mechanoreceptive role. The large peg sensillum in *P. albinotata* shares morphological similarities with the finger-like sensillum observed in *L. coleoptrata*, suggesting potential homology or functional equivalence.

In *M. pandata* (Machaerotidae), the labial tip sensilla comprise five uniporous basiconic sensilla, several peg-like sensilla, and one finger-like sensillum. These sensilla are arranged into two groups that are similar in organisation to those observed in *L. coleoptrata* (Aphrophoridae). In *M. pandata*, however, only basiconic sensilla are present above (BS2) and below (BS1) the stylets, indicating a simpler sensory configuration in these regions.

A comparable arrangement is observed in *P. simulans* (Cercopidae), where the sensilla also form two distinct anterior groups: one comprising five basiconic sensilla and the other containing three basiconic sensilla and one finger-like sensillum. Notably, the distribution of long basiconic sensilla above and below the stylets is consistent with that observed in *M. pandata*, with no deviations reported.

In contrast, species from other families exhibit more variable sensillum distributions. For example, in *Clastoptera* sp. (Clastopteridae), sensilla are scattered rather than grouped in an organised fashion. The anterior sensory field contains four peg-like sensilla, four basiconic sensilla, a multiporous finger-like sensillum presumed to be olfactory, and a smooth, dome-shaped sensillum of thermo-hygroreceptive function. This less organised arrangement suggests a divergence from the pattern observed in the Aphrophoridae, Cercopidae, and Machaerotidae families.

*Eicissus* sp. (Epipygidae) has a configuration of four basiconic sensilla, two coeloconic sensilla, and one finger-like sensillum. As with the aforementioned taxa, no variation was observed in the distribution of basiconic sensilla above or below the stylets. However, the presence of coeloconic sensilla marks a notable deviation, as these are absent in the other species discussed.

In some Membracoidea species, a reduction in the number of sensilla on the labial tip has been observed. For instance, *H. coagulata* (Cicadellinae) has just one pair of mechanosensilla positioned immediately beneath the stylet fascicle exit point [13]. These mechanoreceptors often come into direct contact with the stylets and are not accompanied by chemosensory sensilla, which is considered an atypical configuration for *H. coagulata*. Most other auchenorrhynchn species have at least one porous receptor at the labial tip [51,53]. Leopold et al. [13] proposed that the functional need for chemosensory organs at the labial tip may be reduced in generalist phytophagous species such as *H. coagulata*. In contrast, our study found that other Cicadellinae species have a complete set of chemosensilla on the labial tip. The Cicadellidae species examined—*B. fulviventris*, *C. viridis*, *N. modulatus*, and *S. bicolor*—all displayed six to seven uniporous peg-like sensilla (PS2) and typically one basiconic sensillum (BS3), which were arranged within a distinct semicircular sensory field at the labial tip. A prominent, multiporous, finger-like sensillum, likely serving an olfactory function, was also consistently observed. The distribution of one to three pairs of basiconic sensilla (BS1 and BS2) above and below the stylets was stable across species, except in *C. viridis*, which deviated from this pattern. Additionally, in *C. viridis,* a single long trichoid sensillum was present in the anterior sensory field.

Zhai et al. [14] reported several short Sb1 sensilla adjacent to long trichoid sensilla (St3) on the ventral side of the labial groove in *Nacolus tuberculatus* (Walker) (Hylicinae). Based on our classification, the Sb1 sensilla correspond to PS2, and the St3 sensilla align with BS1. However, no pores were observed in the sensilla of N. tuberculatus, leading Zhai et al. [14] to conclude that these structures are likely to be mechanoreceptive only. By contrast, *B. fulviventris*, which belongs to the same subfamily, exhibits uniporous pegs and basiconic sensilla that serve a gustatory function.

A comparative analysis of *N. modulatus* and *S. bicolor* alongside other Deltocephalinae species, including *Stirellus indrus*, *Alobaldia tobae,* and *Maiestas dorsalis*, reveals a shared sensillar arrangement [17]. These species have eight longer basiconic sensilla (Sb1) and one shorter sensillum (Sb2) on the labial tip. The Sb1 sensilla are homologous to the PS2 sensilla, while the shorter Sb2 sensillum corresponds to the multiporous, finger-like sensillum observed in N. modulatus and *S. bicolor*. These structures are thought to act as receptors for detecting host plant compounds and possibly performing mechanosensory functions.

Zhao et al. [24] reported similar sensillar configurations in *P. striatus* (Deltocephalinae), identifying several short basiconic sensilla (Sb1) and a single long trichoid sensillum at the labial apex. These correspond to the peg (PS2) and trichoid (TS) sensilla in our classification.

A distinct, rounded sensory field containing nine sensilla was observed in *D. japonicus* (Errhomeninae): five uniporous peg-like sensilla (PS2), three uniporous basiconic sensilla (BS3), and one large multiporous finger-like sensillum. One BS1 and one BS2 sensillum were present above and below the stylets, respectively.

A markedly higher number of sensilla were recorded in *Myerslopia* sp. (Myerslopidae): ten peg-like sensilla and one finger-like sensillum were arranged in a circular sensory field. As in other species, one basiconic sensillum was located above (BS2) and below (BS1) the stylets. This unusually high number of peg sensilla may reflect a unique sensory adaptation associated with a specialised feeding strategy or ecological niche.

In *C. corticina* (Ulopidae), the sensory field on the labial tip was narrow and positioned more laterally with five uniporous basiconic sensilla (BS3). In *S. nova* (Membracidae), three BS3 sensilla and two coeloconic sensilla (CS) were identified. A similar configuration was found in *Aetalion* sp. and *Melizoderes* sp., which also belong to the superfamily Membraciodea. BS1, BS2, and TS sensilla were clearly visible on the ventral labial surface of these species.

Various shapes of uniporous gustatory sensilla have been observed on the apex of the labium in many planthoppers (Fulgoromorpha). The most common types include peg sensilla (PGSU1 and PGSU2), clavate sensilla (CLSU), and less commonly, peg-in-pit sensilla (PPSU) [50]. Additionally, five types of multiporous sensilla without flexible sockets have been confirmed: cupola-shaped, oval plate, peg, and complex peg sensilla, all with olfactory function.

As these types of multiporous sensilla are generally considered to have an olfactory function [50,55,59,60,61,62], it has been suggested that the labium may assist the antennae with olfactory perception. Our data on the studied species of Cicadomorpha confirm the possibility of olfaction on the labium. This function is performed by finger-like sensilla.

Among Cicadomorpha, long basiconic sensilla (BS1) are the second most commonly observed sensillum type on the labial tip, while trichoid sensilla are comparatively rare. BS1 sensilla are typically located in the posterior sensory field beneath the maxillary and mandibular stylets, while the sensilla BS2 are usually located above. We hypothesized that this function is involved in contact chemoreception and mechanoreception. These structures may aid in the positioning of the labium during feeding and therefore serve as dual-function receptors. Similar interpretations have been proposed by Backus and McLean [27], Walker and Gordh [63], and Rani and Madhavendra [64], who noted the placement of these sensilla at the labial tip across various hemipteran taxa. Their consistent positioning and morphology suggest a conserved role in host surface probing and plant tissue assessment. Notably, Leopold et al. [13] demonstrated that in *H. coagulata*, the two long basiconic sensilla—referred to as Sb I and Sb II—located at the end of the third labial segment function predominantly as mechanoreceptors.

In the study of the labium sensilla, it was uncertain whether the sensilla basiconica (BSN1 and BSN2)—the second most abundant sensillum type observed on the posterior sensory field on the labial tip in Fulgoromorpha—possess terminal pores, although all are embedded in flexible sockets [50]. Nevertheless, distinguishing between these two types of sensilla remains challenging and often depends on the detection of a terminal pore. These sensilla were generally located in the ventral sensory field, above and beneath the maxillary and mandibular stylets, and likely play a role in assisting with labium positioning during feeding. Foster et al. [51] and Backus [20] suggested that such sensilla should be classified as mechano-chemoreceptive in leafhoppers. Morphological evidence from other hemipteran taxa suggests that contact-chemoreceptive sensilla are relatively common in such areas of the labium in hemipteran species [65,66,67,68,69,70,71].

However, current research has revealed peculiar variations in the number of sensilla in the anterior field. The number of sensilla appears to vary between species (no inter-individual variation was observed). Some species of Membracoidea (*B. fulviventris*, *C. viridis*, *N. modulatus*, *S. bicolor,* and *Myerslopia* sp.), Cercopidea (*P. simulans*), and Cicadoidea (*T. crinita*) are characterised by the presence of a single type of peg sensillum. These sensilla vary in length from 2 to 40 µm and are categorised as either PS1 (longer) or PS2 (shorter). The longest sensilla were found in *P. insignis* (SA: 43 µm), with most oscillating around 6–18 µm. The smallest peg sensilla (SA: 2.1 µm) were observed in *Myerslopia* sp., which had the largest number of sensilla (10) of all the species examined. Some Mebracoidea (e.g., *C. viridis*, *D. japonicus,* and *C. corticana*), Cercopoidea (e.g., *M. pandata* and *Clastoptera* sp.), and Cicadoidea (e.g., *P. insignis*) species had two types of sensillum: peg and basiconica. In these species, the peg sensilla (PS2) were usually more numerous than the basiconica (BS3), although in *M. pandata,* they were equally numerous (four of each). Basiconic sensilla evidently dominated some Membracoidea species (*S. nova*, *Aetalion* sp., and *Melizoderes* sp.) and Cercopoidea species (*L. coleoptrata* and *Eicissus* sp.). The longest BS3 sensilla (SA: 24 µm) were found in *Aetalion* sp., while in the remaining species, they were between 10 and 18 µm long. The multiporous olfactory sensillum (FLS) is common to most of the studied species, except for four species in which the sensilla could not evidently be recognised based on morphological characteristics. Other smaller sensilla include the coeloconica sensilla, found only in three species, and the dome-shaped sensillum, present only in one species.

The posterior field was less differentiated. We usually identified one sensillum basiconicum (BS2) and one to three sensilla basiconica (BS1). The sensillum trichodeum (TS) was extremely rare, only being identified in a few species.

This diverse set of labial tip sensilla undoubtedly provides interesting evolutionary insights for further taxonomic and phylogenetic Cicadomorpha studies.

From a phylogenetic perspective, it is interesting to consider whether the observed types of sensillum are representative and autapomorphic of the groups in which they were found.

Does the differentiation of sensilla in the anterior field into pegs (PS) and basiconica (BS) in the dorsal sensory field represent a defining feature of Cicadomorpha families? Will the three observed patterns of sensillum division and distribution be largely confirmed after examining a larger number of samples? What implications will the presence or absence of an olfactory sensillum on the labial tip have for Cicadomorpha phylogeny with a larger dataset? Is there a relationship between this morphology, the number of sensilla, and diet?

### 4.3. The Cross-Section of the Stylets

This morphological study of mouthparts sheds new light on the structural complexity and diversity of the feeding apparatus in different Cicadomorpha families. The arrangement of the maxillary and mandibular stylets and their interlocking mechanisms, consisting of two locks is highly similar. Slight differences in the size of the food and salivary canals demonstrate conserved features.

However, variations in the structure that closes the salivary canal, such as the hooked process found in cicadellid species and the T-shaped process found in Membracidae (e.g., *S. nova*) and Cicadidae, Cercopidae, and Aphrophoridae suggest that subtle adaptations have occurred in response to specific feeding behaviours. For example, species that feed on phloem or xylem may require different structural reinforcements to regulate pressure and seal the salivary canal. The locks’ structural configuration, consisting of upper and lower hooked and straight processes, ensures the stylets are tightly aligned and stable during penetration and feeding. Similar interlocking mechanisms have been observed by Forbes and Raine [23], Brożek and Herczek [29,30], and Brożek and Bourgoin [50]. The consistency of these mechanisms across multiple taxa indicates their evolutionary importance for efficient fluid feeding.

Mandibles show both symmetrical and asymmetrical configurations across taxa. In most leafhoppers, the mandibles are symmetrical; however, in the Ledrinae, Iassinae, Typhlocybinae, and Membracidae, asymmetry is more prevalent. This suggests that mandibular morphology may serve as a taxonomic character potentially relating to feeding styles. The presence or absence of complete mandibular encasement around the maxillae also reflects varying mechanical demands during stylet penetration. The ability of the mandibles to slide along the maxillae adds to their mechanical flexibility, which is crucial for stylet insertion and movement within plant tissues.

The diagonal configuration, in which the locks turn to the left relative to a perpendicular axis, is a rare condition observed only in two subfamilies: Ledrinae and Iassinae. Emeljanov [34] describes a similar case in some Ledridae and Cicadellidae, where the maxillae were twisted by 45 degrees. This orientation suggests a degree of morphological plasticity that may influence feeding mechanics. The most common state appears to be the perpendicular arrangement of the maxilla in Cicadellidae (Ulopinae, Macropsine, Aphrodinae, Typhlocybine, Cicadellinae, and Deltocephalinae), characterised by dorsal and ventral locks aligning along a vertical axis.

In contrast, the horizontal orientation, in which both locks lie within the internal concavity of the mandibles, is observed in Cicadidae, Tettigarctidae, Cercopidae, Aetalionidae, Myerslopiidae, and Membracidae. Similarly, Emeljanov [34] indicated that, in certain Cercopidae and Cicadidae species, the maxillary stylets twist 90 degrees. This configuration may facilitate greater integration or compactness within the stylet–mandible complex, potentially enhancing piercing efficiency or protecting the locks during rest. The presence of this configuration in multiple taxa suggests that it may be an example of functional adaptation driven by similar ecological pressures.

From a phylogenetic standpoint, the orientation of the dorsal and ventral locks could be a valuable characteristic for cladistic analysis. The rarity of the diagonal type, coupled with the broader distribution of the perpendicular and horizontal types, suggests that this feature may have evolved stepwise. Mapping these traits onto a molecular phylogeny could help to establish whether the orientations are homologous or homoplastic traits within the group.

## 5. Conclusions

This study reveals substantial differences in the morphology of labial sensilla and the structure of stylets among Cicadellidae and other Cicadomorpha taxa, with implications for taxonomy and functional morphology. The consistent presence of a prominent, finger-like olfactory sensillum on the labial tip may serve as a diagnostic feature for cicadomorphans and is likely linked to host plant detection and probing behaviour.

The variation in the number and shape of mandibular stylet barbs indicates adaptations to different host plant tissues. Meanwhile, the conserved dual interlocking system of the maxillary stylets, comprising distinct dorsal and ventral locks, provides structural stability during feeding and represents a fundamental evolutionary trait across Cicadomorpha (autapomorphy), in contrast to the three-interlocking system of the maxillae in remaining Hemiptera. The two types of salivary canal closures observed (hooked and T-shaped) may correspond to feeding specialisation, such as xylem feeding. Comparative analysis among Cicadomorpha families suggests that similarities in sensillum configuration within families such as Aetalionidae, Membracidae, and Melizoderidae may indicate shared evolutionary origins. Similarly, the strong morphological resemblance between the families Myerslopiidae and Cicadellidae suggests a closer evolutionary relationship, although exceptions such as the species C. corticina highlight the need for further taxonomic research. Furthermore, the dorsoventral flattening and relative orientation of the maxillary and mandibular stylets—especially the diagonal arrangement observed in Ledrinae and Iassinae—underscore their potential as phylogenetically informative characters.

In summary, both labial sensillum architecture and stylet morphology are valuable systems for investigating the systematics, evolution, and function of Cicadomorpha. Future studies integrating behavioural, ecological, and ultrastructural approaches are essential to fully understand the adaptive significance and evolutionary pathways of these feeding structures.

## Figures and Tables

**Table 1:** List of 33 studied species.

Superfamily	Family	Species
Cicadoidea	Tettigarctidae	*Tettigarcta crinita* Disant, 1883 *Proarna insignis* Distant, 1881
	Cicadidae	*Cicadetta podolica* (Eichwald 1830)
Cercopoidea	Aphrophoridae	*Lepyronia coleoptrata* (Linnaeus, 758) *Aphrophora costalis* Matsumura, 1903
	Cercopidae	*Prosapia simulans* (Walker, 1858)
	Clastopteridae	*Clastoptera* sp. Germar, 1839
	Epipygidae	*Eicissus decipiens* Fowler, 1897
	Machaerotidae	*Machaerota pandata* Distant, 1916
Membracoidea	Melizoderidae	*Melizoderes* sp. Spinola, 1850
	Myerslopiidae	*Myerslopia* sp. Evans, 1947
	Aetalionidae	*Aetalion* sp. Latreille, 1810
	Membracidae	*Stictopelta nova* Goding, 1892
		*Tolania* sp. Stål, 1858
	Ulopidae	*Ulopa reticulata* (Fabricius, 1794)
		*Coloborrhis corticina* Germar, 1850
	Cicadellidae	*Diodontophorus japonicus* (Ishihara, 1957)
		*Stirellus bicolor* (Van Duzee, 1892)
		*Balala fulviventris* (Walker 1851) *Ledra aurita* (Linnaeus, 1758) *Cicadella viridis* (Linnaeus, 1758) *Nephotettix modulatus* Melichar, 1912 *Neotituria kongosana* (Matsumura, 1915) *Idiocerus stigmaticalis* Lewis, 1834 *Psammotettix alienus* (Dahlbom, 1850) *Iassus lanio* (Linnaeus, 1761) *Macropsis fuscinervis* (Boheman, 1845) *Strogglyocephalus agrestis* (Fallén, 1806) *Kyboasca bipunctata* (Oshanin, 1871)
		*Edwardsiana gratiosa* (Boheman, 1852) *Eupteryx vitatta* (Linnaeus, 1758) *Streptanus sordidus* (Zetterstedt, 1828) *Doratura stylata* (Boheman, 1847)

**Table 2:** The length of the labial tip sensilla (mm/µm) was measured based on the images in Figures 10 and 11 using the measurement tool in CorelDRAW 2019. Each sensillum was measured twice using a horizontal and vertical measuring device. The numbering of the sensilla (1) ect. follows what is shown in Figures 10 and 11. Scale bars in figures are in µm. SA—Arithmetic mean.

	PS2	BS3	FLS	CS	PS1	DP	BS1	BS2	TS
species	mm/ µm	mm/ µm	mm/ µm	mm/ µm	mm/ µm	mm/ µm	mm/ µm	mm/ µm	mm/ µm
*Balala fulviventris*	**(1)** 5.4/11.7 **(2)** 7.4/16.0 **(3)** 13.8/30 **(4)** 9.4/20.0 **(5)** 8.2/17.0 **(6)** 8.6/18.6 **SA=** **8.7/18.8**	-----	**(7)** 3.2/6.9	-----	-----	-----	**(8)** 16.8/36.5 **(9)** 15.8/34.4 **(10)** 16.6/33.9 **SA=** **16.4/34.9**	Not observed	**(11)** 44/95
*Cicadella viridis*	**(1)** 4.0/6.8 **(2)** 4.0/6.8 **(3)** 4.0/6.8 **(4)** 4.3/6.9 **(5)** 6.7/11.0 **(6)** 5.8/9.4 **SA=** **4.8/7.9**	**(7)** 3.5/6.0	**(8)** 9.7/36.3	-----	-----	-----	-----	-----	**(9)** 12/45
*Nephotettix modulatus*	**(1)** 15.0/11.2 **(2)** 21.0/15.7 **(3)** 13.0/9.7 **(4)** 14.4/10.8 **(5)** 11.0/8.2 **(6)** 12.3/9.2 **(7)** 11.0/8.2 **SA=** **13.9/10.4**	-----	**(8)** 7.8/5.8	-----	-----	-----	**(9)** broken (not measured)	-----	**(10)** broken (not measured)
*Stirellus bicolor*	**(1)** 4.9/8.6 **(2)** 7.3/12.8 **(3)** 7.1/12.5 **(4)** 4.9/8.6 **(5)** 4.9/8.6 **(6)** 7.2/12.4 **(7)** 7.4/12.9 **(8)** 2.6/4.5 **SA=** **5.7/10.1**	-----	**(9)** 2.8/4.9	-----	-----	-----	**(10)** broken (not measured)	**(11)** 1.9/3.3	**(12)** broken (not measured)
*Diodontophorus japonicus*	**(1)** 9.3/7.9 **(2)** 9.0/8.2 **(3)** 7.6/6.5 **(4)** 5.1/4.3 **(5)** 6.7/5.7 **(6)** 6.5/5.5 **SA=** **7.3/6.3**	**(7)** 11.2/9.6 **(8)** 9.1/10.7 **(9)** 14.3/12.5 **SA=** **1.5/10.9**	**(10)** 12.1/10.3	-----	-----	-----	**(11)** (not measured) (Figure 10E)	**(12)** 7.7/21	-----
*Myerslopia* sp.	**(1)** 6.8/2.0 **(2)** 8.2/2.4 **(3)** 7.8/2.3 **(4)** 9.5/2.8 **(5)** 9.0/2.6 **(6)** 6.4/1.9 **(7)** 8.4/2.5 **(8)** 7.5/2.2 **(9)** 8.5/2.5 **(10)** 10.1/3.0 **SA=** **8.2/2.1**	-----	**(11)** 18/5.2	-----	-----	-----	**(13)** broken (not measured) (Figure 5C)	**(12)** 16.5/8.7	-----
*Coloborrhis corticina*	**(1)** 5.7/5.0 **(2)** 4.7/4.1 **SA=** **5.2/4.5**	**(3)** 15.2/13.5 **(4)** 10.4/9.2 **(5)** 8.4/7.4 **SA=** **11.3/10.3**	-----	-----	-----	-----	**(6)** 6.7/5.9	**(7)** 4.4/3.8	**(8)** 17.0/25
*Stictopelta nova*	-----	**(1)** 11.8/9.6 **(2)** 16.2/13.2 **(3)** 13.4/11.0 **(4)** 12.1/9.9 **(5)** 10.7/8.7 **SA=** **12.8/10.4**	-----	-----	-----	-----	**(6)** 12.7/30.0	**(7)** 7.3/6.0	-----
*Aetalion* sp.	-----	**(1)** 8.8/18.0 **(2)** 11.0/23.0 **(3)** 15.0/31.0 **SA=** **8.6/24.0**	-----	**(4)** 1.0/2.1 (5) 1.5/3.1	-----	-----	**(6)** 11.8/25.0	**(7)** 10.3/22	**(8)** 18.0/38
*Melizoderes* sp.	-----	**(1)** 7.0/16.0 **(2)** 5.4/12.7 **(3)** 7.0/16.0 **SA=** **6.4/14.9**	-----	**(4)** 1.0/2.3 **(5)** 2.4/5.6	-----	-----	**(6)** 9.6/22.5	**(7)** 6.2/14.5	**(8)** 8.3/19.5
*Lepyronia coleopterata*	-----	**(1)** 16.6/17.3 **(2)** 15.5/16.3 **(3)** 12.7/13.3 **(4)** 12.2/12.8 **(5)** 12.6/13.3 **(6)** 16.0/16.8 **(7)** 19.5/20.5 SA= **15.0/15.7**	**(8)** 11.2/12	-----	-----	-----	**(9)** 13.0/49.0 **(10)** 6.5/24.7	-----	**(11)** 25.0/95.0
*Machaerota pandata*	**(1)** 8.9/6.6 **(2)** 8.9/6.6	**(3)** 29.1/21.8 **(4)** 29.1/21.8 **(5)** 15.1/11.2 **SA=** **24.0/18.2**	**(6)** 11.2/8.4	-----	-----	-----	-----	**(7)** 29.5/22	**(8)** 22.5/44
*Prosapia simulans*	**(1)** 3.0/9.2 **(2)** 3.0/9.2 **(3)** 3.0/9.2 **(4)** 3.0/9.2 **(5)** 3.5/10.7 **(6)** 3.0/9.2 **(7)** 2.8/8.5 **(8)** 4.8/14.5 **SA=** **3.2/9.9**	-----	**(9)** 10.3/7.3	-----	-----	-----	**(10)** 10.2/30.0 **(11)** 10.2/30.0	**(12)** broken (not measured)	-----
*Eicissus* sp.	-----	**(1)** 19.9/10.4 **(2)** 19.3/10.1 **(3)** 42.0/22.1 **(4)** 42.9/22.1 **SA=** **30.0/16.1**	**(7)** 14.2/7.4	**(5)** 4.7/2.4 (6) 4.5/2.3	-----	-----	-----	-----	-----
*Clastoptera* sp.	**(1)** 4.5/7.3 **(2)** 4.6/7.4 **(3)** 4.2/6.8 **(4)** 5.6/9.0 **SA=** **4.7/7.6**	**(5)** 11.8/19.0 **(6)** 6.5/10.5 **(7)** 9.0/14.6 **(8)** 9.0/14.6 **SA=** **9.0/14.6**	**(9)** 5.4/8.7	-----	-----	-----	**(11)** 9.3/15.0 **(12)** 7.7/12.0	**(13)** 4.2/6.8	-----
			**(10)** 1.0/2.6 Dome shaped sensi-llum						
*Tettigarcta crinita*	-----	-----	**(7)** 6.9/10.8 **(8)** 8.6/13.5	-----	**(1)** 14/22 **(2)** 7.2/11 **(3)** 7.8/12 **(4)** 8.9/14 **(5)** 7.6/12 **(6)** 8.9/14 **SA=** **9.0/17.8**	-----	-----	**(9)** 4.4/10	-----
*Proarna insignis*	-----	-----	**(5)** 6.7/8.3 **(6)** 4.6/5.7	**(7)** 3.6/4.5	**(1)** 32/40 **(2)** 37/46 **SA=** **34/43**	**(3)** 9.7/12 **(4)** 9.7/12	**(8)** 20.3/64 **(9)** 12.7/40	**(10)** 7.3/23	-----

## Data Availability

The original contributions presented in the study are included in the article, further inquiries can be directed to the corresponding author.
